# Preventing children’s care entry: an umbrella review of the risks, protective factors, and interventions

**DOI:** 10.1016/j.childyouth.2026.109112

**Published:** 2026-09

**Authors:** Mark Rodgers, Yanhua Chen, Davara Bennett, Michelle Black, Mia Hankinson, Yiwen Liu, David Marshall, David Taylor-Robinson, Amanda Sowden

**Affiliations:** a Centre for Reviews and Dissemination, University of York, York YO10 5DD, United Kingdom; b Institute of Population Health, University of Liverpool, United Kingdom; c School of Medicine, University of Liverpool, United Kingdom

**Keywords:** Care entry, Care re-entry, Looked after children, Risk and protective factors, Interventions

## Abstract

**Background:**

Existing reviews have documented a range of factors associated with care entry or re-entry for children, with much less attention given to the intervention literature. In addition, there has been no systematic attempt to bring these two bodies of evidence together. In this overview of systematic reviews we aimed to identify and synthesise evidence on both risk/protective factors and interventions for preventing care entry/re-entry and to identify potential entry points for policy intervention.

**Methods:**

We identified systematic reviews related to children’s care entry/re-entry, in high-income countries, published since 2013. Selection and data extraction were performed in duplicate, and AMSTAR-2 was used to appraise review quality. We combined findings using narrative synthesis, categorising factors into non-modifiable or modifiable (using the Dahlgren-Whitehead model of health determinants), and linking interventions with these factors where appropriate.

**Results:**

Of the 33 systematic reviews included, 15 focused on risk/protective factors and 18 on interventions. Modifiable factors and related interventions were categorised into individual and lifestyle factors (maternal mental ill-health/substance misuse, child health), social and community factors (social support, child maltreatment), and living and working conditions (socioeconomic and service-level factors). This overview highlighted the multifaceted risk and protective factors associated with children’s entry and re-entry into care, and the interacting and cumulative effect of risk factors. Reviews on the effectiveness of interventions reported limited or inconsistent evidence, often due to different study designs (experimental vs observational), small numbers of included primary studies, or the heterogeneity of interventions. Individual programmes rather than structural policies tended to be the focus in reviews, and most programmes were conducted in the US.

**Conclusions:**

Factors associated with care entry/re-entry are multifaceted and involve complex interactions between upstream and downstream drivers. A range of interventions have been evaluated, but robust evaluations of interventions focusing on context-specific policies are needed to better understand and improve outcomes for children and families.

## 1 Background

Out-of-home care for children is an acute child welfare system intervention, whereby a child or young person who is unable to live with their birth family is accommodated in alternative settings. In the UK, the motivating policy context for this review, the phrase ‘Children looked after’ (CLA) refers to children who are under the care of their local authority for over 24 h, (“Looked after children”) including foster, kinship or residential placements. (NSPCC Learning, 2025). CLA rates in England have risen substantially over the past decade, reaching 81,770 children in care by March 2025 (“Reporting year 2025: Children looked after in England including adoptions”, 2025). Generally, children will cease to be “looked after” when they are either adopted, return home, or reach their 18th birthday (NSPCC Learning, 2025, UK Government). In 2022–23, 27% of children leaving care returned home and were reunited with their birth family within the time-frame of that year (Ford, 2024). Reunification rates can range from 20% to 80% depending on the definition of reunification and differences in placement and setting (Sitjes-Figueras, Maneiro, Garcia-Molsosa, & López-López, 2025). Unfortunately, not all children who return home remain at home. Unsuccessful reunification, leading to care re-entry is a major concern, (Carnochan, Rizik-Baer, & Austin, 2013) as it disrupts continuity of care and may harm health and social development (Semanchin Jones & LaLiberte, 2017). Re-entry also increases the likelihood of subsequent placement disruptions (Finster and Norwalk, 2021, Finster and Norwalk, 2021).

CLA are frequently exposed to significant early adverse experiences, particularly within the family, such as domestic abuse, parental mental health problems, and parental substance misuse. In the UK, the most recent statistics show that two-thirds (66%) of CLA entered care due to abuse or neglect, with the remaining third recorded under other categories, including illness or disability, family relationship breakdown, community violence and exploitation, and forced displacement (such as unaccompanied children seeking asylum) (Education, 2024). These adverse experiences often co-occur, often in challenging socioeconomic contexts, (Adjei et al., 2022, Rod et al., 2020) leading to complex interactions of upstream and downstream drivers (for example, underlying structural causes can contribute to more obvious proximal problems such as neglect). Previous research has identified a wide range of factors that are relevant to both care entry and re-entry, spanning macro-level community and environmental contexts, neighbourhood and household circumstances, family relationships, and individual parent and child characteristics (Opoku et al., 2025, Semanchin Jones and LaLiberte, 2017, Simkiss et al., 2013, Wulczyn et al., 2020). For re-entry, features of the care experience itself and the support provided during reunification have also been highlighted (Ahn et al., 2025, Goldacre et al., 2022, Mc Grath-Lone et al., 2017, Shaw, 2021).

Poor outcomes for care-experienced families and the financial strain on local authorities in England have driven research into modifiable factors and preventive interventions in the UK (Harris et al., 2019, Bennett et al., 2022, McHale et al., 2024). Evidence from the US suggests that financial support for families can reduce maltreatment, care entry, and costs (Rostad, Ports, Tang, & Klevens, 2020). Similarly, US caseworker surveys emphasise that support services can aid successful reunification (Cole and Caron, 2010, Jedwab et al., 2018) family engagement, and monitoring (Cole and Caron, 2010, Farmer, 2014, Frimpong-Manso et al., 2022, Jedwab et al., 2018) However, significant gaps exist. A 2023 England-wide survey of local authorities with children’s services responsibilities found that, among 75 responding local authorities, over half (56%) reported that they did not have a reunification policy or strategy, and 71% reported limited access to specialist support services, which may hinder their statutory duties under the Children Act 1989 to safeguard children's welfare(Learning, 2024). A care plan for the child is a statutory requirement, (“The Care Planning, Placement and Case Review (England) Regulations 2010″, 2010) but beyond general guidance about reunification being considered when it's in the best interests of the child, there is no strong framework for achieving reunification (The Children Act 1989 guidance and regulations, 2021).

To develop effective prevention policies there is a need to understand risk and protective factors, their underpinning mechanisms, the effects of corresponding interventions, and the context in which policies are being considered. While previous research has catalogued these factors, it often fails to explore how they operate or relate to preventive measures (Mc Grath-Lone et al., 2017; Opoku et al.; Simkiss et al., 2013). Building on our scoping review that identified a lack of integrated evidence, (Bennett et al., 2025) we synthesise findings on care (re-)entry factors and related interventions to address the following questions.

• What are the factors for entering or re-entering care?
• What interventions have been found to be effective in preventing care entry or re-entry?

To answer these questions, we followed the Cochrane methods for undertaking Overviews of Reviews, (Higgins et al., 2024) and reported the findings according to the Preferred Reporting Items for Systematic Reviews and Meta-Analyses (PRISMA) statement (Page et al., 2021). Additionally, we interpreted the findings within a UK policy context.

## 2 Methods

### 2.1 Search strategy

Relevant reviews were selected from those included in a scoping review relating to the health of care experienced people (“The Health of Care Experienced People”). The full search strategy can be found in Appendix C. To ensure that the evidence identified is applicable and relevant to the current context, the searches were limited to 2013 onwards.

### 2.2 Study selection

Table 1 describes the eligibility criteria used to select relevant systematic reviews from the map.

**Table 1:** Evidence selection criteria.

	**Inclusion criteria**	**Notes and exclusion criteria**
**Participants**	Children in high-income countries who are not currently in care but are at risk of entering care, or children who have been reunified with family and are at risk of re-entering care	Reviews focused on children who are already in care were excluded
**Risk and protective factors**	Factors that increase or decrease the likelihood of children going into care or re-entering care	−
**Interventions**	Any intervention to prevent care entry or re-entry	−
**Comparators**	Any or none	−
**Outcomes**	Care entry or re-entry reported as an outcome	Other information relating to health outcomes (physical health, mental health, health behaviours or healthcare access and usage including patient experience and quality of care) were extracted from included reviews where relevant
**Study design**	Systematic reviews of empirical primary research of any study design, including qualitative, experimental and observational studies	Reviews did not have to be described as a ‘systematic review’ by authors but must have included a systematic search, inclusion criteria and some form of synthesis. Realist reviews were eligible if they followed systematic methods
**Language**	English language publications	Non-English language publications

### 2.3 Data extraction and quality assessment

The data extraction form was developed collectively by the authors and piloted on a small sample of reviews. All data were extracted by one reviewer in a spreadsheet and checked by a second.

Data extracted included:

· Objective
· Range of contexts (including countries and settings) in which risk factors were identified and/or interventions were delivered
· Outcomes related to care (re)entry
· Relevance to current UK context
· Factors identified as increasing, decreasing, or having no impact on care (re-)entry
· Intervention details (where relevant)
· Intervention impacts on care (re-)entry and health outcomes
· Factors affecting intervention effectiveness or implementation

Reviews were appraised using the Assessment of Multiple Systematic Reviews (AMSTAR-2) tool (Shea et al., 2017).

### 2.4 Synthesising findings

**Fig. 1:**
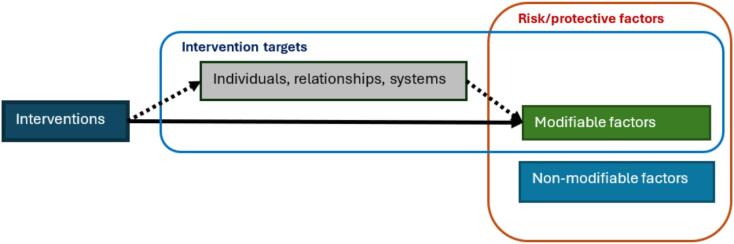
Conceptual diagram of synthesis.

Review findings were combined using narrative synthesis. Risk and protective factors were categorised into modifiable or non-modifiable factors, and modifiable factors were subsequently organised into individual and lifestyle factors, social and community factors, and living and working conditions, based on the Dahlgren-Whitehead model of health determinants (Dahlgren & Whitehead, 2021). The evidence on which factors increase or decrease the risk of entering care was summarised, alongside any inconsistencies or uncertainties in this evidence. Where possible interventions were linked to modifiable factors either directly, or indirectly through other targets (see Fig. 1), by constructing intervention/factor matrices, and we documented any explicit descriptions of mechanisms that were available from the reviews. As well as effectiveness outcomes, contextual factors that could impact effectiveness or implementation of interventions were extracted where reported and used to interpret the effectiveness data. Finally, the evidence was interpreted in light of UK policy.

## 3 Findings

**Fig. 2:**
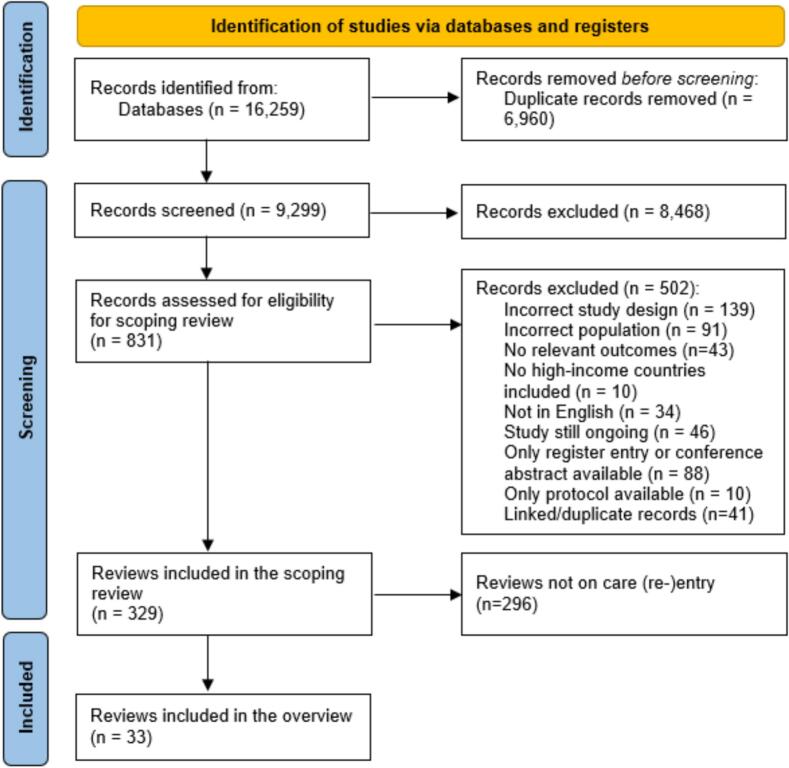
PRISMA flow chart.

Using the search strategy in Appendix C, we identified 16,259 records across different databases, of which 9,299 records were screened, and 329 reviews included in the earlier scoping review. These were assessed for eligibility based on the selection criteria (Table 1), and 33 systematic reviews were included in this overview (296 records were excluded as they did not focus on care entry or re-entry) (Fig. 2).

### 3.1 Quality of evidence

The majority of reviews (N = 27) were judged to be critically low in confidence using AMSTAR-2 (defined as having “…more than one critical flaw in the review and should not be relied on to provide an accurate or comprehensive summary of the available studies”). Four reviews (Bergin et al., 2023, Bezeczky et al., 2020, Economidis et al., 2023, Sheehan, et al., 2018) were deemed to be low in confidence (defined as having “…one critical flaw and may not provide an accurate or comprehensive summary of the available studies”) and two (NICE, 2021, Nurmatov et al., 2020) were moderate in confidence (defined as having “…more than one non-critical weakness but no critical flaw, and may provide an accurate summary of the available studies”). See Appendix B for ratings on AMSTAR-2 for each review.

The most common critical flaws in these reviews were: not providing a list of excluded studies with reasons for their exclusion (N = 29), not considering risk of bias when interpreting the results of the review (N = 24), and not having a protocol specifying the methods to be followed (N = 20). Only three reviews reported evidence separately by study design, (Economidis et al., 2023, Neo et al., 2021, Nurmatov et al., 2020) despite observational studies being more likely to show an effect of interventions reducing care (re-)entry compared to experimental studies. Although it is important to note that the number of experimental studies was small, we also identified methodological issues among these studies, for example a high risk of bias, and small sample size. Within broader-scope reviews, programmes and outcomes were heterogeneous, which may have contributed to the mixed findings regarding the effects of interventions. Evidence for some interventions came from the same few primary studies (e.g., recovery coaches, mentoring programmes), or the reviews focused on outcomes other than care (re-)entry, and the number of primary studies that assessed care (re-)entry was very small (Economidis et al., 2023).

In terms of effectiveness outcomes, the evidence is mixed and inconclusive (see below). Lack of robust primary evaluations is a major limitation of the review literature, which limits understanding of effectiveness in different contexts.

### 3.2 Review characteristics

The reviews included primary study evidence from Argentina, Australia, Belgium, Canada, Denmark, Finland, France, Greece, Israel, Netherlands, New Zealand, Norway, South Africa, Sweden, UK and USA with most studies originating from the USA.

The reviews focused on risk/protective factors for care (re-) entry (N = 15) or interventions/policies to prevent care (re-)entry (N = 18). Risk/protective factors have been categorised as non-modifiable (demographic factors, parental experience of care) or modifiable factors (individual and lifestyle factors, social and community factors, and living and working conditions) using the Dahlgren-Whitehead model of health determinants (Dahlgren & Whitehead, 2021). Interventions have been grouped and matched with their targeted modifiable risk factor(s) where possible. Reviews focusing on care entry, re-entry, or both are presented separately in Table 2 but are synthesised together due to overlapping factors and the number of reviews that assessed both care entry and re-entry. A summary of evidence from reviews of interventions can be found in Table 3.

**Table 2:** Matrix of risk/protective factors and interventions for care (re-)entry from systematic reviews (N = 33).

	**Class of factors**		**Risk/protective factor reviews**				**Intervention reviews**			
			**Outcome examined**			**Mechanisms**	**Outcome examined**			**Mechanisms**
			***Care entry***	***Care re-entry***	***Both***		***Care entry***	***Care re-entry***	***Both***	
**Non–** **modifiable**	**Demographic factors**; **Parental experience of care/history of trauma**		Jaggi (2022) † Schaub (2022) † Simkiss (2013) †	Goodwin (2020) † Semanchin Jones (2017) † Landers (2016) † Lloyd (2018)† White (2016)†	Davidson (2019) † Noorishad (2020) †	Davidson (2019) † Goodwin (2020) † Jaggi (2022) † Semanchin Jones (2017) † Schaub (2022) † Noorishad (2020) †	N/A			N/A
**Modifiable**	**Individual and lifestyle factors**	**Maternal mental health /substance misuse**	Scott (2013) Simkiss (2013) †	Semanchin Jones (2017) † Landers (2016) † Lloyd (2018)†	Davidson (2019) † Wood (2022) †	Landers (2016) † Davidson (2019) †	Neo (2021)	LaBrenz (2020)† NICE (2021) † Zhang (2018)	Meindl (2019)	LaBrenz (2020) † Meindl (2019) Neo (2021) Zhang (2018)
		**Child health factors**	Scott (2013) Simkiss (2013) †	Goodwin (2020) † Semanchin Jones (2017) † Landers (2016) †	Davidson (2019) †	No mechanisms described	−	NICE (2021) †	Bergin (2023)	No mechanisms described
	**Social and community factors**	**Social support**	Jaggi (2022) †	Semanchin Jones (2017) † Lloyd (2018)†	Davidson (2019) †	Jaggi (2022) †	−	LaBrenz (2020)† Saeteurn (2022) †	Stabler (2022)	LaBrenz (2020) † Saeteurn (2022) † Stabler (2022)
		**Child maltreatment/ family dysfunction**	Scott (2013)	Carlson (2020) ^±^ Goodwin (2020) † Semanchin Jones (2017) † Landers (2016) †	Davidson (2019) †	Carlson (2020) ^±^ Davidson (2019) † Landers (2016) †	Bezeczky (2020) † Economidis (2023) † Lee (2014) †	LaBrenz (2020) † NICE (2021) †	Stabler (2022)	Bezeczky (2020) † Economidis (2023) † Lee (2014) † NICE (2021) †
	**Living and working conditions**	**Socioeconomic factors**	Bai (2022) † Jaggi (2022) † Scott (2023) Simkiss (2013) †	Semanchin Jones (2017) † Landers (2016) † Lloyd (2018)† White (2016)†	Davidson (2019) † Noorishad (2020) † Wood (2022) †	Davidson (2019) † Landers (2016) † Lloyd (2018)†	Holdroyd (2023) †	−	Stabler (2022) Wood (2022) †	Holdroyd (2023) † Stabler (2022) Wood (2022) †
		**Service-level factors**	Gregory-Wilson (2022) † Jaggi (2022)† Mason (2019) ^±^	Carlson (2020) ^±^ Goodwin (2020) † Semanchin Jones (2017) † Landers (2016) † White (2016)†	Davidson (2019) †	Carlson (2020) ^±^ Davidson (2019) † Goodwin (2020) † Gregory-Wilson (2022) † Mason (2019) ^±^ White (2016)†	Sheehan (2018)	Lindner (2023) † NICE (2021) †	Nurmatov (2020) † Stabler (2022)	Lindner (2023) † Nurmatov (2020) † Sheehan (2018) Stabler (2022)
	**Cumulative risks**		Simkiss (2013) †	−	Davidson (2019) † Marcellus (2017) †	Marcellus (2017) † Davidson (2019) †	van Assen (2020) †	−	−	van Assen (2020) †

† Reviews that exclusively or predominantly included US studies ^±^Reviews that exclusively or predominantly included UK studies.

**Table 3:** Summary of interventions evidence on care (re-)entry.

	**Intervention target**	**Reviews**	**Intervention names**	**Summary of evidence**	**Overall confidence in the results of the review (AMSTAR-2 ratings)**
**Individual and lifestyle factors**	Maternal mental health/substance misuse	LaBrenz et al. (2020) Meindl and et (2019) Neo et al. (2021) NICE (2021) Zhang et al. (2018)	FTDC/FDAC; Recovery Coaches; Other integrated treatment programmes	A few primary studies showed the effect of FTDC/FDAC on reducing care re-entry/increasing reunification, but *meta*-analysis showed no effect (large heterogeneity); other programmes had mixed findings (non-RCTs more likely to show an effect than RCTs); some degree of overlap in primary studies between reviews	Mostly critically low apart from NICE (2021) which was rated as moderate
	Child health factors	Bergin et al. (2023) NICE (2021)	Infant mental health interventions; Behavioural management interventions	Very little primary evidence and findings were mixed. Other outcomes such as infant mental health/developmental delay were also reported.	Low (Bergin) or moderate (NICE)
**Social and community factors**	Social support	LaBrenz et al. (2020) Saeteurn et al. (2022) Stabler et al. (2022)	Mentoring (Iowa Parent Partner approach)	Small evidence base showing reduced care re-entry at 12 months but not at 24 months (evidence from 1 primary study across 2 reviews).	All critically low
	Child maltreatment/ family dysfunction	Bezeczky et al. (2020) Economidis et al. (2023) Lee et al. (2014) NICE (2021) LaBrenz et al. (2020) Stabler et al. (2022)	Intensive family preservation/reunification services; Multisystemic therapy; Parental training interventions	Mixed evidence (small number of RCTs/non-RCTs found reduced care entry in families that received multisystemic therapy or parental training; short-term effect of intensive preservation services but not at 3 + months), other outcomes such as reduced maltreatment allegations were also reported.	Three critically low (Lee, LaBrenz, Stabler), two low (Bezeczky, Economidis), one moderate (NICE)
**Living and working conditions**	Socioeconomic factors	Holdroyd et al. (2023) Stabler et al. (2022) Wood et al. (2022)	Working tax credits; Family income-change interventions; Welfare reforms	Working tax credits and an increase in family income decreased care entry; benefit reduction increased care entry in non-RCT but not in RCT studies	All critically low
	Service-level factors	Lindner and Hanlon (2023) NICE (2021) Nurmatov et al. (2020) Sheehan et al. (2018) Stabler et al. (2022)	Shared decision-making meetings; Signs of Safety; Policy change; Matching services; Practice/structure change, service integration/coordination, supervision	Mixed or no evidence (due to the limited evidence base with mostly qualitative evidence). Non-RCTs are more likely to suggest a beneficial effect of shared decision-making than RCTs.	Two critically low (Lindner, Stabler), one low (Sheehan), two moderate (NICE, Nurmatov)
Cumulative risks		van Assen et al. (2020)	Multi-domain home visiting interventions	No evidence of reduction in care entry. Other outcomes such as emotional/behavioural problems were also reported.	Critically low

These reviews included children considered to be at risk of care entry (children of care alumni, children from ethnic minorities or Indigenous populations, minoritised gender/sexual orientations), families at risk of their children entering care or whose children are in care and are pursuing reunification (mothers with substance misuse disorders), children currently in care (looked after children < 17 years, infants removed from birth), adoption or foster care families, and reunified children or families at risk of care re-entry. Reviews of population-level interventions were eligible if care entry or re-entry was the outcome evaluated.

### 3.3 Modifiable risk and protective factors for care (re-)entry

**Fig. 3:**
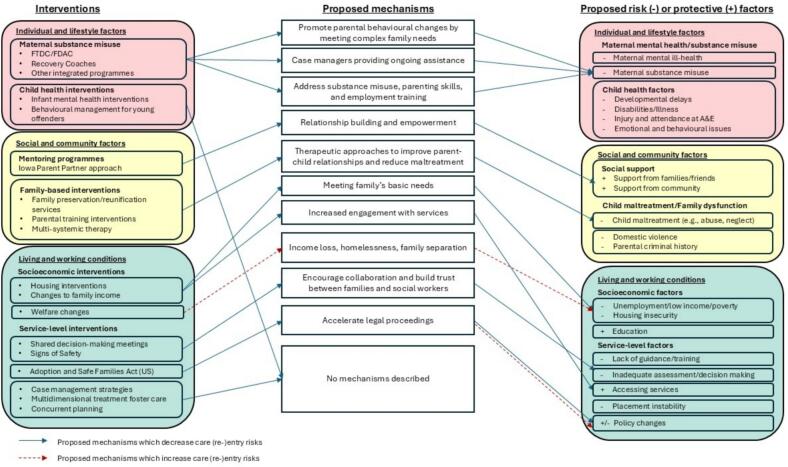
Summary of interventions to prevent care (re-)entry, their proposed mechanisms, and potential impact on risk/protective factors.

The following sections focus on risk and protective factors for care entry or re-entry, grouped into six categories, with an additional section on cumulative risks. Within each section, the impact and mechanisms of risk/protective factors (as reported by the review authors) are described first, followed by a summary of the intervention literature and the proposed mechanisms of these interventions (as described by review authors). A summary of these interventions, their proposed mechanisms (as proposed by review authors), and the risk/protective factors that may be targeted by these interventions is visualised in Fig. 3.

#### 3.3.1 Individual and lifestyle factors

##### 3.3.1.1 Maternal mental ill-health and/or substance misuse

###### 3.3.1.1.1 Risk/protective factors

Seven reviews reported maternal mental ill-health or substance misuse risk factors for care entry, (Scott et al., 2013, Simkiss et al., 2013) re-entry, (Landers and Danes, 2016, Lloyd, 2018, Semanchin Jones and LaLiberte, 2017) or both (Davidson et al., 2019, Wood et al., 2022).

*Impact of risk/protective factors*

All seven reviews (Davidson et al., 2019, Landers and Danes, 2016, Lloyd, 2018, Scott et al., 2013, Semanchin Jones and LaLiberte, 2017, Simkiss et al., 2013, Wood et al., 2022) reported parental substance misuse as a risk factor for care entry or re-entry (following a breakdown in reunification). While none of the reviews quantified this risk, it was considered particularly raised in instances of severe substance misuse (Lloyd, 2018).

Four reviews (Landers and Danes, 2016, Lloyd, 2018, Simkiss et al., 2013, Wood et al., 2022) reported that parental mental ill-health (depression, anxiety, or prior mental health treatment) was a risk factor for children entering public care, and was negatively associated with reunification. The definition of mental illness varied considerably between studies (Simkiss et al., 2013) and across reviews, and effect sizes varied from small to large (Lloyd, 2018).

*Mechanisms described*

Two reviews (Davidson et al., 2019, Landers and Danes, 2016) described potential mechanisms related to maternal mental ill-health and/or substance misuse. Children of parents with substance misuse are more likely to experience abuse and neglect, as well as being exposed to domestic violence, (Davidson et al., 2019) which are additional risk factors for care entry and re-entry (see section on child maltreatment). Parental substance misuse or mental ill-health may shift the balance between family resources and the increasing demands placed on families (Landers & Danes, 2016). One review cited the diathesis-stress model to explain how substance misuse may be considered as a vulnerability which can interact with additional environmental stressors (e.g., socioeconomic stress, lack of support) to increase the risk of care entry and re-entry (Davidson et al., 2019).

###### 3.3.1.1.2 Interventions targeting maternal mental ill-health and/or substance misuse

Five reviews (NICE, 2021, LaBrenz et al., 2020, Meindl et al., 2019, Neo et al., 2021, Zhang et al., 2018) evaluated interventions targeting maternal substance misuse in preventing care (re) entry, with most of the evidence focused on care re-entry. No reviews specifically evaluated interventions for maternal mental ill-health (although this was sometimes included as a part of multi-component interventions).

The population in these reviews included biological parents of children in care (including parents with substance misuse), children in need of care or who have been in care, and looked after children transitioning to live with their adoptive or biological families.

*Family Treatment Drug Courts (FTDC)*.

All reviews (NICE, 2021, LaBrenz et al., 2020, Meindl et al., 2019, Neo et al., 2021, Zhang et al., 2018) evaluated FTDC, which is a court-based intervention in the US offering therapeutic support alongside judicial monitoring. There are different models of FTDC, such as integrated, parallel, dual-tracked or mixed. The integrated model provides one judge who could oversee both the child protection and substance use components of a case, and provides regular court appearances, parenting classes, group and individual counselling, educational support, as well as outpatient substance use rehabilitation services (LaBrenz et al., 2020, Meindl et al., 2019) This integrated model was adapted in the UK and named as Family Drug and Alcohol Courts (FDAC) (Meindl & et, 2019). Similar models have also been implemented in Australia and proposed for South Africa (Gedye, Holness, & Parker, 2026).

FTDC/FDAC aims to provide an alternative model focusing on family functioning by meeting the family’s complex needs (e.g., psychiatric support, employment, housing), which are often present in mothers with substance misuse (LaBrenz et al., 2020, Neo et al., 2021, Zhang et al., 2018). These complex needs are also associated with increased risk of child maltreatment (Neo et al., 2021). Integrated approaches such as the FTDC/FDAC promote parental behavioural change in areas that expose their children to significant harm (Meindl & et, 2019). There are two proposed pathways in reducing care (re-)entry: by creating internal change and increasing engagement in treatment, and creating behaviour change through treatment (Meindl & et, 2019).

Most of these interventions were evaluated in observational studies, and there was little evidence for the effectiveness of FTDC or FDAC in reducing care re-entry (LaBrenz et al., 2020, Zhang et al., 2018). One review (LaBrenz et al., 2020) found that only two of the five included observational studies showed an impact of FTDC on reducing care re-entry (LaBrenz), and another *meta*-analysis (Zhang et al., 2018) with five observational studies found no differences on care re-entry (OR = 0.82, 95% CI = 0.29, 2.28, I-squared = 70.4%). Two reviews reported some evidence for increased reunification, one for FTDC (OR = 1.75, 95% CI = 1.38, 2.22) (Zhang et al., 2018) and one for FDAC (OR = 3.40, 95% CI = 1.07, 10.84 or OR = 2.07, 95% CI = 0.87, 4.91), depending on the definition of reunification durability (NICE, 2021). Another review only described mechanisms of the FDAC model and did not evaluate effectiveness (Meindl & et, 2019).

The different types of FTDC evaluated in these reviews may explain some of the inconsistency in findings (Zhang et al., 2018). Additional factors at the individual court-level (e.g., training and dedication to the programme), local authority level (e.g., inter-agency working, long waiting list and service-related limitations) and policy level (e.g., fit within existing legislation and processes) may also enable or hinder its implementation and effectiveness (Meindl & et, 2019).

*Other substance misuse interventions*.

Other maternal substance misuse interventions include the Recovery Coaches programme, which was evaluated in two reviews (NICE, 2021, LaBrenz et al., 2020) and integrated/multidimensional treatment programmes which were evaluated in one review (Neo et al., 2021). Recovery coaches work as case managers for parents and assist parents with assessments, advocacy, outreach, and case management, and continue to provide home visits after reunification until case closure (LaBrenz et al., 2020). Integrated treatment programmes include multiple components that address substance misuse as well as other maternal needs. They include interventions such as the FTDC/FDAC, as well as other family therapy-based programmes that were adapted to address substance misuse, parenting skills, and employment training (Neo et al., 2021).

Evidence on the recovery coaches programme was inconsistent across the two reviews depending on the outcome assessed. One review (NICE, 2021) (based on one study) found no effect of the programme on foster care re-entry at 1 year follow-up, but the same study also showed an increased likelihood of achieving stable reunification. This same study also appeared in another review (LaBrenz et al., 2020). This suggests that although the programme may be beneficial for reunification stability, it does not have an impact on unstable reunification (i.e. with re-entry into care), but reasons for this were not discussed. Other substance misuse treatment programmes also showed mixed findings on care entry (Neo et al., 2021). Non-RCTs were more likely to show an effect (OR = 0.35, 95% CI = 0.23, 0.52; I-squared = 47%, n = 2,061) compared to RCTs (OR = 0.62, 95% CI = 0.33, 1.18; I-squared = 0%, n = 134), and other than care entry, integrated treatment programmes also increased substance use treatment completion and reduced illicit drug use and alcohol consumption (Neo et al., 2021).

#### 3.3.2 Child health factors

##### 3.3.2.1 Risk/protective factors

Six reviews reported the child’s physical (developmental delay disabilities/illness, injury and attendance at A&E) or mental health (emotional or behavioural issues) as risk factors for care (re-) entry, of which two examined care entry, (Scott et al., 2013, Simkiss et al., 2013) three on care re-entry, (Goodwin and Madden, 2020, Landers and Danes, 2016, Semanchin Jones and LaLiberte, 2017) and one which included both (Davidson et al., 2019).

###### 3.3.2.1.1 Impact of risk/protective factors

Children with physical or mental health problems are disproportionately represented in child welfare systems, (Davidson et al., 2019) although there was very little evidence reported on child health risk factors for care entry and re-entry. Emotional and behavioural problems in children have been reported to contribute to adoption breakdown (Goodwin & Madden, 2020) and care re-entry after reunification, (Semanchin Jones & LaLiberte, 2017) although with inconsistent findings, possibly due to the moderating influence of other factors such as age (Landers & Danes, 2016).

###### 3.3.2.1.2 Mechanisms described

No reviews described any mechanisms related to child health factors.

##### 3.3.2.2 Interventions targeting child health

Two reviews (NICE, 2021, Bergin et al., 2023) evaluated interventions specifically targeting mental health or behavioural problems in children (although this may have been included as a part of multi-component interventions).

The population in these reviews included infants (0–5 years) and their parents, as well as looked after children (<18 years) transitioning to live with adoptive or biological parents.

One review evaluated infant mental health interventions targeting emotional, social, and behavioural indicators for infants, as well as the infant-parent relationship, (Bergin et al., 2023) and another review evaluated behavioural management for young offenders (NICE, 2021). Both of these reviews reported inconsistent evidence on the effectiveness of these interventions in preventing care entry or increasing the likelihood of reunification (NICE, 2021, Bergin et al., 2023).

#### 3.3.3 Social and community factors

##### 3.3.3.1 Social support

###### 3.3.3.1.1 Risk/protective factors

Four reviews reported social support as a protective factor for care (re-)entry, or conversely, a lack of social support as a risk factor for care (re-)entry. One review examined care entry, (Jaggi et al., 2022) two examined care re-entry, (Lloyd, 2018, Semanchin Jones and LaLiberte, 2017) and one included both outcomes (Davidson et al., 2019).

*Impact of risk/protective factors*.

Social support was defined broadly across the reviews, such as placement with relatives, (Davidson et al., 2019) support from family or peers, (Jaggi et al., 2022) or social support at a community-level (Semanchin Jones & LaLiberte, 2017). Although inadequate parental social support was described as being associated with increased care re-entry (Semanchin Jones & LaLiberte, 2017) or decreased likelihood of reunification, (Lloyd, 2018) the magnitude of this risk was not reported in these reviews. Social support was also described as a protective factor, but the impact of this on care entry was not evaluated (Jaggi et al., 2022).

*Mechanisms described*.

One review described a potential mechanisms of social support on care entry. Social support is integral to breaking the cycle of maltreatment among parents with prior experience of care (Jaggi et al., 2022). Familial resources (emotional, practical, or financial support) and peer networks may also improve parenting skills and reduce parental stress by helping parents work through their past (Jaggi et al., 2022).

###### 3.3.3.1.2 Interventions targeting social support

Three reviews (LaBrenz et al., 2020, Saeteurn et al., 2022, Stabler et al., 2022) evaluated mentoring or peer programmes as a source of parental social support. The population in these reviews included biological parents of children in care, in need of care, or who have been in care.

Mentoring programmes were the focus in three reviews, although only two reviews (LaBrenz et al., 2020, Saeteurn et al., 2022) reported on their effectiveness, and one scoping review (Stabler et al., 2022) mapped the mentoring programmes found in the literature. Both reviews evaluated the same peer parent programme (Iowa Parent Partner approach), which is an intervention that matches parents with veteran parents with experience of the child welfare system, as a way of providing personalised support and guidance (LaBrenz et al., 2020, Saeteurn et al., 2022). Parents were matched based on similar characteristics or needs, including mental health problems or substance misuse (LaBrenz et al., 2020).

Mentoring support is theorised to work by building a relationship between the parent and someone with similar experiences, and is often provided as part of a larger programme of work (Stabler et al., 2022). This relationship is thought to provide parents with resources that can empower and motivate them to meet the needs of their children when they return home (Saeteurn et al., 2022). It also provides opportunities to engage with social workers, lawyers, and other community services (e.g., mental health) to receive additional support as needed (LaBrenz et al., 2020). These resources may then minimise the likelihood of their children re-entering the care system (Saeteurn et al., 2022).

Both reviews included the same study which reported care re-entry as an outcome, and found that parents who participated in the programme had children who were less likely to re-enter care within 12 months of reunification, but this effect was no longer significant after 24 months (LaBrenz et al., 2020, Saeteurn et al., 2022). These reviews also evaluated other outcomes such as permanency and time in care, (Saeteurn et al., 2022) or focused on other family-based interventions where mentoring was only one of several interventions evaluated, (LaBrenz et al., 2020) therefore overall there was very limited evidence for this programme.

##### 3.3.3.2 Child maltreatment and family dysfunction

###### 3.3.3.2.1 Risk/protective factors

Six reviews reported child maltreatment (abuse, neglect) or family dysfunction (domestic violence, parental criminal history) as risk factors for care (re-)entry, of which one examined care entry,(Scott et al., 2013) four on care re-entry, (Carlson et al., 2020, Goodwin and Madden, 2020, Landers and Danes, 2016, Semanchin Jones and LaLiberte, 2017) and one which included both (Davidson et al., 2019).

*Impact of risk/protective factors*.

Childhood maltreatment increased the risk of care (re-)entry in five reviews, (Carlson et al., 2020, Davidson et al., 2019, Landers and Danes, 2016, Scott et al., 2013, Semanchin Jones and LaLiberte, 2017) in particular for neglect, physical abuse, and sexual abuse (Davidson et al., 2019, Scott et al., 2013, Semanchin Jones and LaLiberte, 2017). Two reviews (Davidson et al., 2019, Scott et al., 2013) reported neglect as the most common reason for entering care. One review (Davidson et al., 2019) reported longer time to reunification or greater rates of care re-entry for children removed due to neglect compared to physical or sexual abuse, although another (Landers & Danes, 2016) showed inconsistent findings for reunification outcomes. This inconsistency may be due to other demographic factors (age and race/ethnicity of children) and the complex nature of neglect, which is often a result of other risk factors such as unemployment or poverty (Landers & Danes, 2016).

Other family dysfunction such as domestic violence and parental criminal history were also reported as risk factors for care entry or a breakdown in placement in three reviews (Goodwin and Madden, 2020, Scott et al., 2013, Semanchin Jones and LaLiberte, 2017). Adoptive parents who lacked therapeutic parenting skills negatively affected post-adoption relationships, although its impact on care re-entry was not reported (Goodwin & Madden, 2020). Domestic violence was also a common reason for care entry, (Scott et al., 2013) and was associated with increased risk of care re-entry along with parental criminal history, (Semanchin Jones & LaLiberte, 2017) although these were only described as risk factors and the magnitude of these risks was not reported.

*Mechanisms described*.

Three reviews put forward potential mechanisms related to child maltreatment/family dysfunction. “Poor parenting” (no definition provided) was highlighted as the greatest predictor for child maltreatment, which may lead to care re-entry in some cases (Carlson et al., 2020). Child maltreatment, in particular neglect, is also associated with socioeconomic risk factors, (Davidson et al., 2019, Landers and Danes, 2016) and is more commonly reported in disadvantaged neighbourhoods. This may be due to environmental stressors (e.g., community violence, fewer daycare providers) and a lack of social support in less socially integrated neighbourhoods, which may influence the way parents interact with their child (Davidson et al., 2019). One review drew upon the diathesis-stress model to show how vulnerabilities in the family (e.g., domestic violence, substance misuse, parenting stress) may also interact with environmental stressors (e.g., lack of support or financial resources) and increase the risk of care entry or re-entry (Davidson et al., 2019).

###### 3.3.3.2.2 Interventions targeting child maltreatment and family dysfunction

Six reviews (NICE, 2021, Bezeczky et al., 2020, Economidis et al., 2023, LaBrenz et al., 2020, Lee et al., 2014, Stabler et al., 2022) evaluated family-based interventions aimed at improving parent–child interactions to reduce care (re-)entry, although these interventions were not directly aimed at reducing child maltreatment, and only one review specifically evaluated family-based therapy on child maltreatment (Economidis et al., 2023).

The population in these reviews varied from children in need of care/have been in care (including children with behavioural problems), families of children at risk of entering care (e.g., due to maltreatment), biological parents of children in care, and looked after children transitioning to live with adoptive or biological parents.

Interventions evaluated in these reviews included intensive family preservation or reunification services, (NICE, 2021, Bezeczky et al., 2020, LaBrenz et al., 2020) multi-systemic therapy, (NICE, 2021, Economidis et al., 2023, Lee et al., 2014) and parental training interventions (NICE, 2021). Intensive family preservation services such as the Homebuilders model was delivered to families with children at risk of placement, and includes key components such as fast response (within 24 h of referral), 24/7 availability, and low caseloads for staff (Bezeczky et al., 2020, Lee et al., 2014). Similarly, intensive reunification services adapt to each individual family’s needs, and include multiple components such as parent–child visitation, group classes, and family therapy (LaBrenz et al., 2020). Other family-based interventions such as multi-systemic therapy aim to promote positive interactions between the youth and their families across multiple life domains (e.g., home, school, peers, community) (Lee et al., 2014) and reduce the risk of child maltreatment, (Economidis et al., 2023) or to provide training and education to parents (NICE, 2021).

These family-based interventions are theorised to work through a combination of therapeutic approaches and education to improve the quality of parent and child relationships (Economidis et al., 2023). Some are based on the crisis intervention theory which proposes that families in crisis are more likely to be motivated to change and are more open to learning new behaviours (Bezeczky et al., 2020). It teaches parents a variety of skills that enable their child to be cared for safely at home and reduce the risk of maltreatment (Economidis et al., 2023, Stabler et al., 2022). Common elements and mechanisms may include problem-solving skills, monitoring, goal setting, and motivational interviewing, (Economidis et al., 2023, Lee et al., 2014) and programmes with an ecological focus were more successful as they are more holistic (Lee et al., 2014).

There was inconsistent evidence for the effectiveness of family-based interventions depending on the type of intervention and evidence evaluated. Evidence from RCTs suggest some reduction in placement risk in families that received multi-systemic therapy (risk difference in percentage points in favour of the intervention group was −0.9 and −17.4), although this was only based on two RCTs, and two observational studies showed inconsistent effects (one in favour of the intervention and one with negligible difference) (Economidis et al., 2023). The beneficial effect of family preservation/reunification services on reducing care entry was only found at one month, and was no longer significant at 3, 6, or 12 months (Bezeczky et al., 2020). RCTs for parental training programmes also found inconsistent evidence on rates of care re-entry and placement breakdown (NICE, 2021). In addition, there was some evidence to suggest that families who received intensive reunification services had fewer maltreatment allegations and referrals to authorities compared to families in the comparison group (LaBrenz et al., 2020). Other reviews either only reported on the effectiveness of individual programme or practice elements, (Lee et al., 2014) or described interventions without assessing their effectiveness (Stabler et al., 2022).

#### 3.3.4 Living and working conditions

##### 3.3.4.1 Socioeconomic factors

###### 3.3.4.1.1 Risk/protective factors

Eleven reviews reported socioeconomic factors as risk factors for (re-)entering care, of which four focused on care entry, (Bai et al., 2022, Jaggi et al., 2022, Scott et al., 2013, Simkiss et al., 2013) four on care re-entry, (Landers and Danes, 2016, Lloyd, 2018, Semanchin Jones and LaLiberte, 2017, White, 2016) and three reviews included both (Davidson et al., 2019, Noorishad et al., 2020, Wood et al., 2022).

*Impact of risk/protective factors*.

The definition of socioeconomic risk factors varied within and between reviews, including caregiver unemployment, family poverty, low income, and lower levels of education. These were associated with increased risk of care (re-)entry in most reviews, (Davidson et al., 2019, Landers and Danes, 2016, Lloyd, 2018, Noorishad et al., 2020, Scott et al., 2013, Semanchin Jones and LaLiberte, 2017, Simkiss et al., 2013, Wood et al., 2022) apart from one (White, 2016), which found full-time employment of the primary caregiver was associated with increased risk of placement discontinuity.

Housing insecurity (e.g., homelessness, emergency housing) also increased the risk of care entry.(Bai et al., 2022, Wood et al., 2022). Children from families with housing insecurity were 4–9 times more likely to enter care compared to families without these experiences (Bai et al., 2022). Neighbourhood disadvantages were also associated with increased risk of child maltreatment (Davidson et al., 2019) and care entry and re-entry, (Noorishad et al., 2020, Semanchin Jones and LaLiberte, 2017) and explained the disproportionate rate of care entry among Black youth even after accounting for deprivation (Noorishad et al., 2020).

Education was described as a protective factor for children of care alumni parents, and parents’ completion of upper secondary school greatly mitigated their children’s risk of care entry (Jaggi et al., 2022). Stable maternal employment, secure housing and increased family income were also described as protective factors for care entry and re-entry (Wood et al., 2022). However, no effect sizes or mechanisms for these protective factors were described in the reviews.

*Mechanisms described*.

Socioeconomic disadvantage is likely to be an important upstream cause in care (re-)entry, (Scott et al., 2013) and housing insecurity and neighbourhood socioeconomic factors were associated with higher likelihood of child maltreatment investigations, (Bai et al., 2022, Davidson et al., 2019) itself a risk factor for care entry and re-entry (see section on child maltreatment).

Three reviews (Davidson et al., 2019, Landers and Danes, 2016, Lloyd, 2018) described potential mechanisms linking socioeconomic factors to care entry and re-entry. Neighbourhood disadvantages can affect collective efficacy (agreeing on what’s acceptable behaviour and holding others accountable), participation in local organisations, access to community resources, and can influence environmental stressors such as community violence (Davidson et al., 2019). These may impact the way parents interact with their child, contributing to higher rates of child maltreatment reports (Davidson et al., 2019). Structural risks like poverty and unemployment can also make it more difficult for mothers with substance misuse to adhere to treatment, reducing the likelihood that mothers will reunify with their children (Lloyd, 2018). The shift in balance between family demands such as poverty and capabilities such as receiving government assistance may also have implications for reunification and was highlighted as an area for future research (Landers & Danes, 2016).

###### 3.3.4.1.2 Interventions targeting socioeconomic factors

Three reviews (Holdroyd et al., 2023, Stabler et al., 2022, Wood et al., 2022) described or evaluated interventions that aimed to increase or decrease a family’s income. The population in these reviews included children and families at risk of care or pursuing reunification.

Two reviews (Holdroyd et al., 2023, Wood et al., 2022) evaluated interventions that changed a family’s income, and one scoping review (Stabler et al., 2022) described available interventions without providing evidence on their effectiveness. The types of interventions evaluated were housing interventions, (Wood et al., 2022) multicomponent interventions that increased a family’s income, (Wood et al., 2022) and welfare changes (e.g., working tax credits, benefit reduction schemes) (Holdroyd et al., 2023, Wood et al., 2022).

Interventions aimed at increasing family income are theorised to work via multiple mechanisms. Economic support can reduce family poverty, leading to improved housing quality, reduced crowding and increased ability for the family to meet basic needs (e.g., provision of food, clothing, and shelter) (Holdroyd et al., 2023, Wood et al., 2022). These in turn may mitigate against other risk factors for care entry and re-entry such as child maltreatment, parental substance misuse, and parental mental ill-health (Wood et al., 2022). In theory, the provision of material help means that families can focus on other services and build trusting relationships with social workers, which encourages engagement with services and reduces the risk of care entry (Wood et al., 2022). On the other hand, welfare changes that reduce a family’s income can lead to income loss, potentially increasing the risk of homelessness, which can trigger family separation or lead to unsafe environments through new co-habitation arrangements (Wood et al., 2022). Although income reduction may prompt mothers to seek employment, low-wage or irregular working hours can reduce child supervision time and increase the risk of neglect unless reliable childcare can be arranged (Wood et al., 2022).

There was some evidence to suggest that working tax credits resulted in a decreased rate of care entry, (Holdroyd et al., 2023) and other interventions that increased the family’s income also showed some evidence of decreasing the risk of care entry or increasing the likelihood of reunification (Wood et al., 2022). Conversely, welfare policies that decreased a family’s income (e.g., benefit reduction) increased care entry or decreased the likelihood of reunification, although this evidence only came from non-RCTs, whereas RCT evidence suggested no effect on care entry (Wood et al., 2022).

Multiple factors may impact the effectiveness of these interventions, such as the amount of tax credit earned, (Holdroyd et al., 2023) family context (e.g., existing debt), discretion or transparency in providing aid (e.g., eligibility, stigma), local housing and labour markets, as well as whether benefit withdrawal was voluntary or punitive (Wood et al., 2022). These factors can affect how income changes are perceived and absorbed, which in turn can affect the relationship between families and social workers and engagement with services (Wood et al., 2022).

##### 3.3.4.2 Service-level factors

###### 3.3.4.2.1 Risk/protective factors

Nine reviews reported service-level factors (lack of guidance/training, inadequate assessment and decision making, policy changes, accessing services, placement instability) for the risk of care (re-) entry, of which three examined care entry, (Claire et al., 2019, Gregory-Wilson et al., 2022, Jaggi et al., 2022) five on care re-entry, (Carlson et al., 2020, Goodwin and Madden, 2020, Landers and Danes, 2016, Semanchin Jones and LaLiberte, 2017, White, 2016) and one which included both (Davidson et al., 2019).

Most of these reviews included qualitative evidence and the effect of service-level factors on rates of care (re-)entry was not reported. A summary of the factors investigated is summarised below.

*Lack of guidance/training*.

Lack of guidance and insufficient training was a theme identified in two reviews, (Carlson et al., 2020, Claire et al., 2019) one on care entry and one on re-entry (which specifically focused on UK evidence), including a lack of guidance on case management, pre-birth assessments, specialist knowledge (e.g., child development or the impact of substance misuse during pregnancy), and poor knowledge of child protection procedures. However, none of these were directly associated with increased risk of care (re-)entry and only qualitative evidence was presented.

*Inadequate assessment/decision-making*.

Issues with the assessment and decision-making process were also identified as factors influencing outcomes in child welfare in two reviews focusing on care entry (Claire et al., 2019, Gregory-Wilson et al., 2022). Lateness and delay with pre-birth assessments were regarded as system-level challenges, (Claire et al., 2019) and health information about the infant and their parents can also influence the court’s decision to remove infants, (Gregory-Wilson et al., 2022) although the impact of these on care entry was not reported.

*Accessing services*.

Accessing services such as parenting classes or mental health resources, including services that link with other risks such as substance misuse were found to be protective in two reviews and may reduce the risk of care entry, (Claire et al., 2019, Jaggi et al., 2022) although another review found inconsistent evidence on care entry (White, 2016). Follow-up support was reported to be insufficient and varied depending on the child’s pathway into care, and support from other agencies led to more successful reunification according to a review which focused on UK evidence (Carlson et al., 2020).

*Placement instability*.

Four reviews (Carlson et al., 2020, Goodwin and Madden, 2020, Landers and Danes, 2016, Semanchin Jones and LaLiberte, 2017) reported placement instability as a risk factor for care re-entry, one of which focused on UK evidence (Carlson et al., 2020). Higher number of placements, shorter placements, and oscillation between care and home were associated with increased likelihood of a breakdown in adoption or reunification, and increased care re-entry (Carlson et al., 2020, Goodwin and Madden, 2020, Landers and Danes, 2016, Semanchin Jones and LaLiberte, 2017).

*Policy changes*.

An additional review described regional and national policy changes as impacting on rates of care entry, reunification and re-entry (Davidson et al., 2019). Policy changes include the Adoption and Safe Families Act of 1997 in the US, which impacted reunification and care re-entry (see intervention section for more information), and failure-to-protect laws, which led to an increase in maltreatment reports due to changes to reasons for removal (Davidson et al., 2019).

*Mechanisms described*.

Six reviews described the likely mechanisms related to service-level factors. A review of UK evidence found that a lack of guidance or inconsistencies in practice may lead to vulnerable children being left in unsafe situations, which could contribute to poor outcomes (Carlson et al., 2020). A lack of trauma-informed training or information about the adopted child may also hinder relationship building between adoptive parents and their child and contribute to adoption breakdown (Goodwin & Madden, 2020). Inadequate assessments do not address mothers’ needs and could be held against them in child protection reports, exacerbating negative experiences and behaviours in mothers (Claire et al., 2019). Conversely, a positive relationship between mothers and staff may be a protective factor, as it acts as a motivating factor and supports access to healthcare services as well as facilitates disclosure of additional needs such as substance misuse (Claire et al., 2019). Policy changes can impact the process of care entry by changing the ways in which maltreatment is reported, as well interactions between children, their families, and their communities, and may have unintended consequences (Davidson et al., 2019). Similarly, some evidence on accessing certain services (e.g., for substance abuse) suggests that there may also be unintended consequences of receiving services, such as setting unrealistic expectations (White, 2016). Finally, different professionals may prioritise different information in predicting the likelihood of abuse (Gregory-Wilson et al., 2022). Professional biases and systematic discrimination may disproportionately affect families from minority backgrounds, contributing to their higher rates of removal (Gregory-Wilson et al., 2022).

###### 3.3.4.2.2 Interventions targeting service-level factors

Five reviews (NICE, 2021, Lindner and Hanlon, 2023, Nurmatov et al., 2020, Sheehan, et al., 2018, Stabler et al., 2022) evaluated service-related interventions, of which one examined care entry, two on re-entry, and two included both outcomes. The population in these reviews included children (and their families) at risk of entering care or already in care and looked after children transitioning to live with adoptive or biological parents.

Various interventions were described or evaluated in these reviews, including shared decision-making meeting service, (Nurmatov et al., 2020, Stabler et al., 2022) Signs of Safety, (Sheehan & et al., 2018) matching or family finding services, (NICE, 2021) policy change, (Lindner & Hanlon, 2023) as well as practice and structure change, service integration/coordination and supervision (Stabler et al., 2022).

*Shared decision-making meetings and Signs of Safety*.

Shared decision-making meetings include the family, child, social worker, and other professionals in developing a plan to maintain child safety and wellbeing, and inform the next steps of social work involvement (Nurmatov et al., 2020, Stabler et al., 2022). The most common service is Family Group Conference (FGC) which is the model used in the UK (Nurmatov et al., 2020). One review (Nurmatov et al., 2020) evaluated its impact on care entry and re-entry and reported mixed results. Non-RCTs were more likely to suggest a beneficial effect of shared decision-making meetings in preventing care entry and re-entry compared to RCTs.

Signs of Safety aims to reduce the number of children in care by drawing on elements of Solution Focused Brief Therapy, which focuses on family strengths and resources and goal setting (Sheehan & et al., 2018). It aims to improve child safety through the development and use of safety plans and networks. One review found insufficient evidence to establish whether or not this approach is effective at reducing care entry, due to the four relevant studies collectively providing very low certainty evidence (Sheehan & et al., 2018).

Both shared decision-making meetings and Signs of Safety are theorised to work by encouraging collaboration between families and social workers. This can build trust, reduce shame, develop shared responsibility for child safety and enable families to participate in important decision making (Nurmatov et al., 2020, Sheehan, et al., 2018).

*Policy change*.

A national policy change (Adoption and Safe Families Act in the US) which aimed to achieve more reunification or placement permanency by accelerating legal proceedings was also evaluated in one review, and although the size of the foster care population declined nationally, it varied between states (Lindner & Hanlon, 2023). Other interventions such as matching services or family finding services, practice/structure change, service integration/coordination and supervision of social workers were only briefly discussed by reviews with little or no evidence provided (NICE, 2021, Stabler et al., 2022).

*Other service-related interventions*.

One review also evaluated other service-level interventions including case management strategies like family searching, multidimensional treatment foster care (MTFC) and concurrent planning (NICE, 2021). There was some evidence from a small number of primary studies showing improvements in rates of permanency (NICE, 2021).

#### 3.3.5 Cumulative risk

##### 3.3.5.1 Risk/protective factors

Three reviews described the impact of cumulative risk factors on care (re-)entry, of which one examined care entry, (Simkiss et al., 2013) and two examined both care entry and re-entry.(Davidson et al., 2019, Marcellus et al., 2017). Cumulative risk factors included socioeconomic factors, maternal substance misuse and mental health, demographic factors such as maternal age, single parenthood, current partner violence, and child health characteristics (e.g., premature birth/low birth weight, infant trauma/illness).

###### 3.3.5.1.1 Impact of risk factors

Although multiple risk factors for care (re-)entry were identified across these reviews, there was often very little evidence for each individual risk factor, and some reviews found that no one specific factor can categorically identify risk of care entry or re-entry (Davidson et al., 2019, Marcellus et al., 2017, Simkiss et al., 2013). An increased number of risk factors was associated with a decreased likelihood of reunification and increased risk of care entry and re-entry, (Davidson et al., 2019) and it was the cumulative impact of risk factors over time that was more important than any risk factor in isolation (Marcellus et al., 2017, Simkiss et al., 2013).

###### 3.3.5.1.2 Mechanisms described

Two reviews (Davidson et al., 2019, Marcellus et al., 2017) described the likely mechanisms related to cumulative risks. From the perspective of a cumulative risk model, multiple risk factors are a greater predictor than individual risk factors (Marcellus et al., 2017). Another review proposed that vulnerabilities for an adverse outcome (e.g., maternal substance misuse for the termination of parental rights) may interact with environmental stressors (e.g, poor socioeconomic circumstances) to trigger care entry and re-entry, consistent with the diathesis-stress model (Davidson et al., 2019). An increased number of risk factors can lead to increased stress, which can impact the family’s ability to engage with and benefit from services, and increase the likelihood of returning to a previous home environment that may lead to care re-entry (Davidson et al., 2019).

#### 3.3.6 Interventions targeting multiple risks

Although most of the family-based interventions can be considered multi-component, they primarily address one area such as substance misuse or parent–child interactions. One review(van Assen et al., 2020) evaluated the effect of multi-domain home visiting interventions that targeted multiple areas of life in families with complex problems on care entry.

Families with complex and multiple problems may experience both socioeconomic difficulties and challenges in care provision and therefore are distinct from families who primarily have parenting or behavioural problems. Multi-domain home visiting interventions provide care in the family’s home environment, provide care workers the opportunity to engage in ecological diagnostics, support them in their daily life and improve relationships with the family. These interventions are characterised by high intensity contacts, small caseloads and flexible care provision. However, evidence from this review (van Assen et al., 2020) suggests that although these interventions may reduce emotional and behavioural problems, there was no reduction in care placements.

##### 3.3.6.1 Non-modifiable risk and protective factors for care (re-entry)

Ten reviews described non-modifiable risk factors for the risk of care (re-)entry, of which three examined care entry, (Jaggi et al., 2022, Schaub et al., 2022, Simkiss et al., 2013) five on care re-entry, (Goodwin and Madden, 2020, Landers and Danes, 2016, Lloyd, 2018, Semanchin Jones and LaLiberte, 2017, White, 2016) and two included both (Davidson et al., 2019, Noorishad et al., 2020).

Specific risk factors reported in these reviews included demographic characteristics (age, sex, race/ethnicity, sexual/gender orientation) and parental history of trauma or out-of-home-care experience.

Findings were mixed and inconsistent regarding the age (Davidson et al., 2019, Goodwin and Madden, 2020, Landers and Danes, 2016, Semanchin Jones and LaLiberte, 2017, Simkiss et al., 2013) or sex (Davidson et al., 2019, Landers and Danes, 2016, Noorishad et al., 2020) of the child. LGBTQ + youths were more likely to have experienced childhood maltreatment and experienced a higher number of placements than non-LGBTQ + counterparts (Schaub et al., 2022). Children from ethnic minority backgrounds were also disproportionately represented in the child welfare system (Davidson et al., 2019, Noorishad et al., 2020) and were more likely to experience placement breakdown, (Goodwin & Madden, 2020) although findings were mixed,(Semanchin Jones & LaLiberte, 2017) non-specific, (Simkiss et al., 2013) or other modifiable factors explained more variance in the outcome (White, 2016). Parental history of trauma was associated with increased risk of care entry and re-entry in one review which also investigated multiple risk factors, (Davidson et al., 2019) and children of parents with prior experience of care were also more likely to enter care compared to the general population (8–19% vs 1% or 3–5 times more likely) (Jaggi et al., 2022).

Although demographic characteristics and parental history of trauma/care experience were considered as non-modifiable factors, some of their mechanisms were considered to be modifiable by review authors. Younger children may require a permanency plan sooner than older children, (Davidson et al., 2019) and adoptive families may prefer infants (Davidson et al., 2019) or there may be an increase in parental stress with older adopted children that may contribute to placement breakdown (Goodwin and Madden, 2020, Semanchin Jones and LaLiberte, 2017). Children from ethnic minority backgrounds may be more likely to be referred to child welfare due to racial biases, (Noorishad et al., 2020) or there may be other mediating factors such as parental substance misuse or socioeconomic status (Noorishad et al., 2020, Semanchin Jones and LaLiberte, 2017). However, racial groups differ in characteristics, making it difficult to identify structural racial biases as the only active factor in decision making (Noorishad et al., 2020). Similarly, LGBTQ + youths may experience familial rejection due to their sexual/gender identity and may also experience homelessness as well as mental health difficulties which may contribute to the risk of care entry (Schaub et al., 2022). Care alumni parents are also more likely to experience socioeconomic and mental health risks among other disadvantages, which can affect their interactions with their children and subsequently increase their children’s risk of care entry (Jaggi et al., 2022).

## 4 Discussion

This overview provides a comprehensive synthesis of the risk/protective factors and associated interventions based on 33 reviews focusing on preventing care entry or re-entry for children. A wide array of factors is associated with children entering and re-entering care. The Dahlgren-Whitehead model of health determinants (Dahlgren & Whitehead, 2021) offers a comprehensive framework for understanding these multilayered factors. Risk/protective factors emerging from the evidence were mapped onto multiple layers of the Dahlgren-Whitehead Model, (Dahlgren & Whitehead, 2021) ranging from outer-layer structural conditions (e.g., socioeconomic adversity), through service environments and family functioning, to individual characteristics. Intervention evidence is mappable at each of these levels.

There are also ‘vertical links’ between the socioeconomic and cultural determinants of care entry and individual and family dynamic factors (Dahlgren & Whitehead, 2021). For instance, the family stress model posits that wider socioeconomic conditions affecting families' material conditions may also increase family stress, leading to poorer mental health, frayed interpersonal relationships and negative coping mechanisms (e.g. substance misuse) (Masarik & Conger, 2017). While many studies have established associations between various risk factors and children’s entry into care, we must distinguish correlation from causation. This overview also highlights cumulative risks, a relatively underexplored area in previous reviews, stressing the fact that multiple adversities co-occur in the lived realities of children in or on the edge of care.

Our synthesis echoes longstanding observations that socioeconomic adversities are upstream drivers of children’s entry into care. This aligns with evidence from a longitudinal ecological study of English local authorities, which found that rising child poverty was associated with higher rates of children entering care, (Bennett et al., 2022) and with prior evidence on potential pathways linking socioeconomic disadvantage, maltreatment, and subsequent care entry. Longitudinal cohort evidence from the UK Millennium Cohort Study shows that over 33% children in the UK experience persistent poverty throughout childhood, increasing risks for poor adolescent health outcomes (Adjei et al., 2022). As there is a consistent and strong association between poverty and child abuse and neglect, child protection practices should address this issue by enabling families, communities, and society to provide environments in which children can thrive (Skinner et al., 2023, Walsh et al., 2019). Systematic review evidence has shown that adversity is more common with social disadvantage. However, it should be noted that emotional abuse is more likely to be identified alongside neglect in contexts of visible material deprivation, whereas it may be under-recognised in more affluent families where signs of harm are less visible. Furthermore, these disadvantages are geographically patterned. In the North of England, for example, over one third of children live in poverty, which is a substantially higher rate than the national average, and the rate at which children enter care is also higher (Pickett, Taylor-Robinson, & Erlam, 2021). Our synthesis also suggests that factors such as parental education, stable employment, secure housing, and income support may function as protective factors against care entry or re-entry; however, quantitative evidence remains limited and predominantly derived from US studies, underscoring the need to strengthen the UK evidence base to better guide policy and practice.

Parental risk factors, particularly maternal mental ill-health and substance misuse, are consistently identified as major drivers of children’s entry to public care. Mental health problems such as depression and anxiety increase the risk of entry, while substance misuse, especially when severe, raises the likelihood of (re-)entry and reduces the chances of reunification. Intervention evidence for substance use mainly covers Family Treatment/Drug and Alcohol Courts, recovery coaches, and integrated treatment programmes, but findings are mixed, study overlap is common, and the evidence base remains weak. Despite growing enthusiasm for FDACs in the UK, robust evidence remains limited. New local evaluations offer opportunities to strengthen the UK's evidence base and emphasise the importance of incorporating evaluation into local authority interventions to ensure sustainability (Davey et al., 2022).

Another family-related factor is child maltreatment and family dysfunction, (Morris, Featherstone, Hood, Bowyer, & Hay, 2022) for which evidence addressing the scale of the problems and the effectiveness of interventions remains extremely scarce, (Gautschi & Lätsch, 2024) as shown in this overview. In addition, neglect is strongly associated with socioeconomic disadvantage (Farooq et al., 2024, Maguire-Jack and Font, 2017, Walsh et al., 2019) and external stressors (e.g., lack of support, financial hardship) may pose greater challenges to families. In line with the family stress model, socioeconomic hardship can heighten parental stress and increase risks of neglect and care entry. For such issues, family-based interventions, including preservation or reunification services, multisystemic therapy, and parental training, have attracted more attention, with the aim of improving family skills to reduce risk. It is essential to clarify, both theoretically and in practice, whose needs are prioritised within “family-focused” interventions, as this is a critical consideration for responding effectively to child maltreatment. In addition, the financial hardship component should not be overlooked.

Social support from relatives, peers, or a community network has been widely recognised as a potential protective factor against care (re-)entry (Saar-Heiman et al., 2024, Saeteurn et al., 2022). A lack of parental social support was described as a risk factor for re-entry and a reduced likelihood of reunification, although the magnitude of these associations was rarely reported. Evidence from related interventions indicates that mentoring or peer programmes, designed as sources of parental social support, aim to provide personalised guidance and to help parents access resources that may empower and motivate them to meet their children’s when they return home. In the UK, emerging models of parent advocacy extend this principle further, embedding parents with lived experience as advocates within local authorities to support and empower other families and shape child protection system change (The Child Welfare Parent Advocacy Movement, 2020, Saar-Heiman et al., 2024).

Service-level factors are diverse, including lack of guidance or training, inadequate assessment and decision making, policy changes, difficulties in accessing services, and placement instability, all of which may influence the risk of care (re-)entry. Interventions span the family level (e.g., Family Group Conferences, FGC) and regional or national levels (e.g., the Adoption and Safe Families Act in the US). However, the evidence base on service-level factors is weak and intervention effects appear highly dependent on context and system conditions. In the UK, for instance, a recent large-scale randomised trial of FGCs (currently at the pre-proceedings stage) found that referral to FGCs reduced the likelihood of children entering care, helping more children remain safely with their families (“Family Group Conferencing at pre-proceedings stage − Foundations”). Beyond questions of effectiveness, FGCs reflect a rights-based approach, which is consistent with the UN Convention on the Rights of the Child. It recognises families' right to participate in decisions that affect them, valuing their role as a long-term resource in the lives of their children.

A salient observation is that child health factors, including both physical health problems (e.g., developmental delay, disability or illness, injury, and emergency department attendance) and mental health difficulties (e.g., emotional or behavioural problems), are associated with an increased risk of care (re-)entry. Evidence suggests that children with such health problems are disproportionately represented among children experiencing child welfare intervention (Allik et al., 2024, Cummings and Shelton, 2024). However, intervention evidence remains scarce. Specific evaluated interventions are limited to infant mental health and young offender behavioural management programmes. Recent evidence shows that children with a social worker are significantly less likely to have their referrals to specialist mental health services, (Morgan et al., 2025) emphasising that universal services are not operating in a proportionate way to meet the needs of this high-risk group. Furthermore, the systematic review evidence identified in this overview did not report any mechanisms for child health factor-related intervention.

In addition to organising emergent factors under the Dahlgren-Whitehead Model, we also emphasise a relatively underexplored aspect in previous research − cumulative risks, which are associated with a decreased likelihood of reunification and an increased risk of care (re-)entry, with their cumulative impact over time proving more important than any single factor in isolation (Font, Sattler, & Gershoff, 2018). As investigated by Rutter (Rutter, 1978) who constructed the first family adversity index based on multiple environmental risk factors for child mental health, the model has since been used to explain various health outcomes. Another model − the diathesis-stress model − also considers the interaction between multiple risk factors (Zuckerman, 1999). More attention should be paid to the accumulation of multiple adversities. For example, previous research using data from a national sample of over 4.2 million social care assessments in the UK has revealed that children entering care are often exposed to complex combinations of risks at home, most frequently domestic abuse, co-occurring with parental mental health issues and, in some instances, substance misuse (Hood et al., 2023). However, socioeconomic adversity is rarely captured within cumulative risk frameworks, limiting their ability to account for structural drivers such as poverty and deprivation. This highlights the importance of considering these broader contextual factors to avoid isolating risks from their social and economic context. Current interventions targeting multiple risks are mostly family-based, such as home visiting interventions that deliver services in the home environment, requiring care workers to conduct ecological assessments, engage in high-intensity contacts, and provide flexible support. However, evidence from one review indicates that while these interventions may reduce children’s emotional and behavioural problems, they do not appear to reduce rates of care placement (van Assen et al., 2020).

### 4.1 Relevance to UK context

Most of these reviews predominantly or exclusively included studies from the US (NICE, 2021, Bai et al., 2022, Bezeczky et al., 2020, Davidson et al., 2019, Economidis et al., 2023, Gregory-Wilson et al., 2022, Holdroyd et al., 2023, Jaggi et al., 2022, Landers and Danes, 2016, Lee et al., 2014, Lloyd, 2018, Marcellus et al., 2017, Noorishad et al., 2020, Nurmatov et al., 2020, Saeteurn et al., 2022, Schaub et al., 2022, Semanchin Jones and LaLiberte, 2017, Simkiss et al., 2013, van Assen et al., 2020, White, 2016, Wood et al., 2022) and some reviewed the impact of policies (e.g., Adoption and Safe Families Act, Fostering Connections to Success and Increasing Adoptions Act) that are specific to the US.(Goodwin and Madden, 2020, LaBrenz et al., 2020, Lindner and Hanlon, 2023). A few reviews attempted to make their findings relevant to the UK by only including evidence from European, North American and Australasian countries, as despite different legal and social frameworks, research from these countries was deemed more likely to be applicable (Claire et al., 2019, Sheehan, et al., 2018, Stabler et al., 2022). Others evaluated intervention models (e.g., FDAC) or intervention components (e.g., concurrent planning, family searching services) that were available in the UK,(NICE, 2021, Meindl et al., 2019, Neo et al., 2021, Zhang et al., 2018) two framed their review evidence in terms of the Scottish system,(Bergin et al., 2023, Scott et al., 2013) and one review limited its evidence to the UK (mostly England) (Carlson et al., 2020). A minority of reviews were concerned with the experience of Indigenous/Aboriginal/First Nations children, which may not easily generalise to a UK context.

Several applicability concerns are reflected in these reviews. Although determinants of health may be similar across countries, some risk factors may be culturally determined and differ between countries, and the organisation and role of health and social services vary widely, making generalisations difficult (Simkiss et al., 2013). Definitions of out-of-home care will also differ across contexts. Variations in standard care across countries can hinder the interpretation of the effectiveness evidence, especially when it is unclear what the comparison group received (NICE, 2021). Differences in legal, cultural and socioeconomic factors may influence how health information is understood (Gregory-Wilson et al., 2022). There may also be differences between countries that implement the same intervention model. For example, in the US, the majority of children are placed in custody during FTDC/FDAC proceedings, whereas in the UK and other countries, there are more occasions where the goal is placement prevention and the child remains at home while the parent is in FDAC proceedings (Meindl & et, 2019).

### 4.2 Strengths and limitations

#### 4.2.1 Strengths

This overview used transparent and rigorous methods to identify, appraise and synthesise evidence. As part of the synthesis, we categorised risk and protective factors for care entry and re-entry into modifiable and non-modifiable factors, and modifiable factors were additionally classified using the Dahlgren-Whitehead model of health determinants (Dahlgren & Whitehead, 2021). Matrices were used to map modifiable factors to intervention targets where possible, highlighting possible entry points for policy intervention. As care entry and re-entry are related but distinct outcomes, the factor matrix distinguished reviews focusing on care entry, care re-entry, or both. This allowed us to examine overlapping factors across outcomes while still identifying factors more specific to care re-entry where reported. Recognising this overlap is also policy-relevant because it may support an integrated approach to prevention and intervention, rather than treating initial entry and post-reunification re-entry as entirely separate policy issues.

Our findings relating to the identification of factors associated with childhood out-of-home care entry and re-entry, are encouragingly consistent with another recently published overview. As our overview was broader in scope and assessed the effects of interventions, we were able to map modifiable factors to intervention targets. Plausible mechanisms as well as contextual considerations (proposed by the review authors) were also incorporated into the synthesis.

#### 4.2.2 Limitations

Although efforts were made to conduct a methodologically rigorous overview, there were some unavoidable limitations. Publication bias may be a potential limitation of this overview. Also, by focusing on previous reviews, rather than the individual primary studies that comprise them, there is a degree of abstraction and loss of contextual detail that is likely to be important when evaluating and interpreting the evidence base. Moreover, the review-level evidence did not always allow a fully separate synthesis of initial entry and subsequent re-entry, and separate analyses may provide clearer insight into outcome-specific risk and protective factors.

The overall quality of the evidence base is limited. Quality appraisal of included reviews using the AMSTAR-2 tool identified a number of methodological weaknesses, with the majority of included reviews rated as “critically low” in confidence (Shea et al., 2017). Methodological reporting and transparency are both set at a high threshold in AMSTAR-2. The most common reasons for low ratings in these reviews were the lack of registered protocols, incomplete reporting of excluded studies, and insufficient consideration of bias. In many intervention reviews, the evidence was derived from a small set of primary studies, which may limit the generalisability of their findings and limit the broader conclusions that can be drawn from the evidence. Reviews included primary studies with different designs which limited the confidence in the findings reported; for example, observational studies were more likely than RCTs to show beneficial effects of interventions on care entry or re-entry, and may be confounded by unmeasured variables. The heterogeneity of interventions limited the generalisability of the findings from reviews, and the inclusion of reviews with mostly US-based evidence raises additional concerns about the applicability of these findings to the UK and other countries, given the legal and cultural differences.

### 4.3 Implications

Policy makers want solutions to tackle care entry and re-entry and to protect children from harm. We provide two main implications, a multifaced approach which enshrines children’s rights and the need for robust evaluation. Reasons for care entry are complex, and consequently effective interventions are difficult to identify and measure. This complexity and scarcity of effectiveness data is reflected in our review findings. What is clear from our review, and consistent with the current evidence base, is that risk factors are multifaceted and socioeconomic hardship and the consequences of it are major risk factors for care entry. The multifaceted nature of the reasons for care entry requires a systemic approach, and embedding children’s rights in legislation and national frameworks may be the broadest reaching approach to alleviating hardship for children, providing foundational legislation which may enhance efforts to reduce care entry.

In terms of what works, the evidence is mixed and inconclusive. The evidence is most consistent for income in the home, inconsistent on interventions targeting maternal mental health/substance misuse, limited for the effectiveness of both social support and family dysfunction/maltreatment in the short term, and limited or inconsistent for service level and child health factors. This does not mean that intervening is of limited value, rather the evidence is insufficiently strong in guiding us on how best to intervene. Lack of robust evaluation is a major limitation of the review literature, which limits understanding of effectiveness in different contexts, and hence should be built into future interventions/policy initiatives to reduce care entry.

It is essential that practice changes are accompanied by rigorous evaluation. For example, the large-scale evaluation of FDAC found that children under FDAC care proceedings were less likely to be placed into Local Authority care (28.6% vs 54.7%) compared to children in standard care proceedings, implying a reduced risk of care entry (Papaioannou et al.). Similarly, the recent Family VOICE trial in England is an important attempt to rigorously evaluate FGCs, a practice that has been widely implemented despite the lack of strong evidence of effectiveness (Scourfield et al., 2024). The evaluation of the Pan-Bedfordshire FDAC pilot highlights its potential in reducing care entry by supporting parents to address substance misuse and stabilise family circumstances (Allen, Paskell, Godar, Ryan, & Clery, 2021). While awaiting further evaluation evidence, it is also essential to adhere to a rights-based approach − as The Promise in Scotland demonstrates − that listens to the needs articulated by children and families and drives meaningful system change alongside the evidence-based agenda (“The Promise Scotland | Transforming how Scotland cares for children, families, and care-experienced adults”).

In addition, better data infrastructure is essential for generating robust intervention evidence. For example, to understand how investment in preventative services affects care entry, a national study of local authority spending in England found that higher investment in family support, children’s centres and youth services was associated with substantially lower rates of children entering care. Between 2014–5 and 2021–22, cuts to such spending accounted for roughly one third of the rise in children looked after (Webb, 2025). Beyond ecological analyses, high-quality administrative data (e.g., ECHILD database (Mc Grath-Lone et al., 2022)) also offers opportunities to evaluate natural policy experiments, as exemplified in decentralised and centralised policy contexts, (Cancian et al., 2017, McLaughlin, 2017, McLaughlin, 2018, Wildeman and Fallesen, 2017) enabling researchers to explore causal impacts of national policy shifts on rates of care entry and re-entry, such as welfare policy changes affecting child-related benefits in the UK (e.g., such as the two-child limit on child-related benefits).

### 4.4 Conclusions

In summary, we have provided a comprehensive overview of the available evidence, taking in modifiable risk factors, policies and strategies to prevent care entry or re-entry, aiming to shed light on the mechanisms underpinning successful prevention and consider the implications for policy. This overview highlights the multifaceted risk and protective factors associated with children’s entry and re-entry into care, and in particular the interacting and cumulative effect of risk factors. To address different risk factors, a range of interventions have been implemented, from family-based programmes to service-level strategies, although the evidence base remains fragmented, weak and highly context dependent. Robust evaluations are scarce, particularly in the UK and other European countries. Overall, preventing unnecessary care entry and re-entry demands both upstream structural actions to tackle poverty and disadvantage, and rigorous, context-sensitive evaluations of interventions to ensure that policies and practices genuinely improve outcomes for children and families.

## Declaration of competing interest

The authors declare that they have no known competing financial interests or personal relationships that could have appeared to influence the work reported in this paper.
