# Supplementary material for: Preventing children’s care entry: an umbrella review of the risks, protective factors, and interventions
Source: Child Youth Serv Rev. 2026 Sep;188:109112. doi: 10.1016/j.childyouth.2026.109112 (PMC13397380; doi:10.1016/j.childyouth.2026.109112)
Supplement: Supplementary file 1 [file mmc1.docx]

# Appendix A Overlap in primary evidence from intervention reviews

Primary studies from the intervention reviews were mapped to investigate the degree of overlap across reviews, which is important to consider as the same studies being included across multiple reviews may lead to an overestimation of the effect of interventions. See below for a visual representation of the overlap in primary evidence.

*Interventions targeting maternal mental ill-health and/or substance misuse*

There was some overlap in primary evidence between the four reviews which reported on the effectiveness of substance misuse interventions. Of the 17 primary studies included across the four reviews on FTDC/FDAC, seven (41%) were included in at least two reviews (one of which was included in three reviews). Only one review focused exclusively on FTDC/FDAC as the intervention, whereas the other reviews looked at a broad set of substance misuse interventions, or focused on interventions other than substance misuse. Only two primary studies on recovery coaches were included in two reviews, and one appeared in both. Out of the four primary studies on integrated treatment programmes other than the FTDC/FDAC, one was included in two reviews. Overall, the percentage of overlap was 39% (9/23 studies appeared in at least two reviews).

###### Interventions targeting child health

There was no overlap in primary evidence between the two reviews, as one focused on infant mental health,^1^ and the other on young offenders, which only included one primary study.^2^

###### Interventions targeting social support

There was no overlap in primary evidence as each review primarily evaluated different types of family-based interventions, and even when two reviews^3,4^ evaluated similar interventions (intensive family preservation/reunification services), one was focused on preventing care entry,^3^ and one on care re-entry.^4^

###### Interventions targeting socioeconomic factors

The types of interventions evaluated in each review were different in focus, and therefore there was no overlap in the primary studies that each included. One review^5^ evaluated a specific form of working tax credits - the Earned Income Tax Credit (EITC) in the US - whereas another^6^ reviewed multiple income change interventions, including housing support, multi-component interventions such as family preservation programmes, and benefit reduction policies.

###### Interventions targeting service-level factors

There was very little overlap in primary evidence as each review primarily focused on a particular type of service-related intervention. Two reviews^2,7^ evaluated the same intervention (shared decision-making meetings), but only one primary study appeared in both, as one review^7^ only included quantitative evidence of children at risk of or are already in care, whereas the other review^2^ included both quantitative and qualitative evidence and exclusively focused on children transitioning out of care.

## Socioeconomic factors

**Primary studies included in reviews of socioeconomic interventions**

| **Primary study** | **Review*** | |
| --- | --- | --- |
|  | **Holdroyd** | **Wood** |
| Berger 2017 |  |  |
| Biehl 2018 |  |  |
| Cancian 2017 |  |  |
| Fein 2003 |  |  |
| Fraser 1996 |  |  |
| Huebner 2012 |  |  |
| Klevens 2017 |  |  |
| Kovski 2022a |  |  |
| Kovski 2022b |  |  |
| Paxson 2003 |  |  |
| Puls 2021 |  |  |
| Rostad 2020 |  |  |
| Ryan 2004 |  |  |
| Shinn 2017 |  |  |
| Thleman 1992 |  |  |
| Thomas 2022 |  |  |
| Wells 2003 |  |  |
| Wells 2006 |  |  |
| Wildeman 2017 |  |  |

*Stabler was excluded as it did not evaluate the effectiveness of interventions

Yellow highlighted cells indicate primary studies included in each review.

## Maternal mental ill-health/substance misuse

**Primary studies included in reviews of maternal substance misuse interventions**

| **Primary study** | **Review*** | | | |
| --- | --- | --- | --- | --- |
|  | **LaBrenz** | **Neo** | **NICE** | **Zhang** |
| ***Variations of FTDC*** | | | | |
| Ashford 2004 |  |  |  |  |
| Boles 2007 |  |  |  |  |
| Brook 2007 |  |  |  |  |
| Bruns 2012 |  |  |  |  |
| Burrus 2011 |  |  |  |  |
| Carey 2010 |  |  |  |  |
| Chuang 2012 |  |  |  |  |
| Gifford 2016 |  |  |  |  |
| Green 2007 |  |  |  |  |
| Harwin 2018 |  |  |  |  |
| Kissick 2015 |  |  |  |  |
| Mackin 2013 |  |  |  |  |
| McMillin 2007 |  |  |  |  |
| Pollock 2015 |  |  |  |  |
| van Wormer 2016 |  |  |  |  |
| Worcel 2008 |  |  |  |  |
| Zeller 2007 |  |  |  |  |
| ***Recovery coaches*** | | | | |
| Ryan 2006 |  |  |  |  |
| Ryan 2016 |  |  |  |  |
| ***Integrated programmes (combining substance use with family-oriented services)*** | | | | |
| Dakof 2009 |  |  |  |  |
| Dakof 2010 |  |  |  |  |
| Donohue 2014 |  |  |  |  |
| Schaeffer 2013 |  |  |  |  |

*Meindl was excluded as it did not evaluate the effectiveness of interventions

Yellow highlighted cells indicate primary studies included in each review.

## Social support

**Primary studies included in reviews of social support interventions**

| **Primary study** | **Review*** | |
| --- | --- | --- |
|  | **LaBrenz** | **Saeteurn** |
| Berrick 2011 |  |  |
| Bohannan 2016 |  |  |
| Chambers 2019 |  |  |
| Enano 2017 |  |  |

*Stabler was excluded as it did not evaluate the effectiveness of interventions

Yellow highlighted cells indicate primary studies included in each review.

## Child maltreatment/family dysfunction

**Primary studies included in reviews of child maltreatment/family-based interventions**

| **Primary study** | **Review*** | | | |
| --- | --- | --- | --- | --- |
|  | **Bezeczky** | **Economidis** | **LaBrenz** | **NICE** |
| ***Parent skills/training interventions*** | | | | |
| Akin 2014 |  |  |  |  |
| Akin 2018a |  |  |  |  |
| Akin 2018b |  |  |  |  |
| DeGarmo 2013 |  |  |  |  |
| Fisher 2005 |  |  |  |  |
| Pasalich 2016 |  |  |  |  |
| Price 2008 |  |  |  |  |
| Spieker 2014 |  |  |  |  |
| Swenson 2000 |  |  |  |  |
| Taussig 2012 |  |  |  |  |
| Trout 2013 |  |  |  |  |
| Trout 2019 |  |  |  |  |
| ***Intensive family preservation/reunification services*** | | | | |
| Berquist 1993 |  |  |  |  |
| Biehal 2005 |  |  |  |  |
| Blythe 2002 |  |  |  |  |
| Brandon 2006 |  |  |  |  |
| Centre for the study of social policy 1998 |  |  |  |  |
| Chambers 2016 |  |  |  |  |
| Ciliberti 1998 |  |  |  |  |
| Daegnais 2003 |  |  |  |  |
| Dennis-Small 1986 |  |  |  |  |
| Feldman 1991a |  |  |  |  |
| Feldman 1991b |  |  |  |  |
| Forrester 2008 |  |  |  |  |
| Forrester 2014 |  |  |  |  |
| Halper 981 |  |  |  |  |
| Hennepin County Community Services Department 1980 |  |  |  |  |
| Jones 1976 |  |  |  |  |
| Jones 1985 |  |  |  |  |
| Kirk 2004 |  |  |  |  |
| Lyle 1983 |  |  |  |  |
| Mitchell 1989 |  |  |  |  |
| Nebraska Department of Public Welfare 1981 |  |  |  |  |
| Pecora 1995 |  |  |  |  |
| Pine 2009 |  |  |  |  |
| Raschick 1997 |  |  |  |  |
| Rubin 1997 |  |  |  |  |
| Schuerman 1994 |  |  |  |  |
| Schwartz 1991 |  |  |  |  |
| Szykula 1985 |  |  |  |  |
| US Department of Health and Human Services 2002 |  |  |  |  |
| Walters 2006 |  |  |  |  |
| Walton 1997 |  |  |  |  |
| Walton 2001 |  |  |  |  |
| Wheeler 1992 |  |  |  |  |
| Willems 1981 |  |  |  |  |
| Wood 1988 |  |  |  |  |
| Yuan 1990 |  |  |  |  |
| ***Family-based therapy (e.g., Multisystemic therapy)*** | | | | |
| Chaffin 2011 |  |  |  |  |
| Chaffin 2004 |  |  |  |  |
| Donohue 2014 |  |  |  |  |
| Foley 2016 |  |  |  |  |
| Kolko 1996 |  |  |  |  |
| Kolko 2018 |  |  |  |  |
| Meezan 1998 |  |  |  |  |
| Runyon 2010 |  |  |  |  |
| Rushton 2010 |  |  |  |  |
| Schaeffer 2021 |  |  |  |  |
| Schaeffer 2013 |  |  |  |  |
| Swenson 2010 |  |  |  |  |
| Thomas 2011 |  |  |  |  |
| Turner 2017 |  |  |  |  |
| Villodas 2021 |  |  |  |  |

*Lee was excluded as it did not evaluate whole interventions and did not provide a list of included studies. Stabler was excluded as it did not evaluate the effectiveness of interventions.

Yellow highlighted cells indicate primary studies included in each review.

## Child health factors

**Primary studies included in reviews of child health interventions**

| **Primary study** | **Review** | |
| --- | --- | --- |
|  | **Bergin** | **NICE** |
| Biehal 2011 |  |  |
| Casanueva 2013 |  |  |
| Casanueva 2019 |  |  |
| Chinitz 2017 |  |  |
| Cyr 2022 |  |  |
| Hazen 2021 |  |  |
| Stacks 2020 |  |  |
| Twomey 2010 |  |  |
| van der Asdonk 2019 |  |  |
| van der Asdonk 2020 |  |  |

Yellow highlighted cells indicate primary studies included in each review.

## Service-level factors

**Primary studies included in reviews of service-level interventions**

| **Primary study** | **Review*** | | | |
| --- | --- | --- | --- | --- |
|  | **Lindner** | **NICE** | **Nurmatov** | **Sheehan** |
| ***Policy - Adoption and Safe Families Act*** | | | | |
| Ashby 2002 |  |  |  |  |
| Graham 2000 |  |  |  |  |
| Green 2006 |  |  |  |  |
| Hansen 2004 |  |  |  |  |
| Hansen 2007 |  |  |  |  |
| Harris-McKoy 2014 |  |  |  |  |
| McDonald 2003 |  |  |  |  |
| McWey 2006 |  |  |  |  |
| McWey 2008 |  |  |  |  |
| Mitchell 2005 |  |  |  |  |
| Naidich 2005 |  |  |  |  |
| Radel 2021 |  |  |  |  |
| Rockhill 2007 |  |  |  |  |
| Smith 2006 |  |  |  |  |
| Spar 2004 |  |  |  |  |
| Townsend 2008 |  |  |  |  |
| Ward 2002 |  |  |  |  |
| White 2003 |  |  |  |  |
| Wulczyn 2002 |  |  |  |  |
| Wulczyn 2006 |  |  |  |  |
| ***Shared decision-making meetings*** | | | | |
| Allan 2015 |  |  |  |  |
| Augsberger 2014 |  |  |  |  |
| Beehlar 2016 |  |  |  |  |
| Berzin 2006 |  |  |  |  |
| Berzin 2008 |  |  |  |  |
| Chambers 2016 |  |  |  |  |
| Crampton 2007 |  |  |  |  |
| Crampton 2011 |  |  |  |  |
| Dijkstra 2016 |  |  |  |  |
| Dijkstra 2018a |  |  |  |  |
| Dijkstra 2018b |  |  |  |  |
| Feldman 2017 |  |  |  |  |
| Godinet 2010 |  |  |  |  |
| Hollinshead 2017 |  |  |  |  |
| Heubner 2012 |  |  |  |  |
| Lambert 2017 |  |  |  |  |
| Mason 2017 |  |  |  |  |
| Munro 2017 |  |  |  |  |
| Onrust 2015 |  |  |  |  |
| Pennell 2000 |  |  |  |  |
| Pennell 2010 |  |  |  |  |
| Perry 2013 |  |  |  |  |
| Sheets 2009 |  |  |  |  |
| Sundell 2004 |  |  |  |  |
| Teal 2013 |  |  |  |  |
| Titcomb 2005 |  |  |  |  |
| Walker 2005 |  |  |  |  |
| Wang 2012 |  |  |  |  |
| Weisz 2006 |  |  |  |  |
| Wheeler 2003 |  |  |  |  |
| Wijnen-Lunenburg 2008 |  |  |  |  |
| YMCA 2014 |  |  |  |  |
| ***Signs of Safety (social work practice)*** | | | | |
| Baginsky 2017 |  |  |  |  |
| Beattie (n.d.) |  |  |  |  |
| Brent Council’s Community and Wellbeing Scrutiny Committee 2017 |  |  |  |  |
| Bunn 2013 |  |  |  |  |
| Caslor 2011 |  |  |  |  |
| City and County of Swansea 2014 |  |  |  |  |
| Department for Child Protection (DCP) 2011 |  |  |  |  |
| Department for Child Protection (DCP) 2012 |  |  |  |  |
| Gardner 2008 |  |  |  |  |
| Gibson 2014 |  |  |  |  |
| Hayes 2012 |  |  |  |  |
| Hayes 2014 |  |  |  |  |
| Holmgard 2013 |  |  |  |  |
| Keddell 2011a |  |  |  |  |
| Keddell 2011b |  |  |  |  |
| Keddell 2013 |  |  |  |  |
| Lohrbach 2004 |  |  |  |  |
| Lwin 2014 |  |  |  |  |
| Munro 2016 |  |  |  |  |
| Nelson-Dusek 2015 |  |  |  |  |
| Reekers 2018 |  |  |  |  |
| Rijbroek 2017 |  |  |  |  |
| Roberts 2016 |  |  |  |  |
| Roberts 2018 |  |  |  |  |
| Rodger 2017 |  |  |  |  |
| Rothe 2013 |  |  |  |  |
| Salveron 2015 |  |  |  |  |
| Skrypek 2012 |  |  |  |  |
| Sorensen 2018 |  |  |  |  |
| Stanley 2014 |  |  |  |  |
| Stanley 2018 |  |  |  |  |
| Turnell 2007 |  |  |  |  |
| Vink 2017 |  |  |  |  |
| Westbrock 2006 |  |  |  |  |
| ***Family searching services*** | | | | |
| Boel-Studt 2017 |  |  |  |  |
| Feldman 2016 |  |  |  |  |
| Landsman 2014 |  |  |  |  |
| ***Multidimensional Treatment Foster Care*** | | | | |
| Castellanos-Brown 2010 |  |  |  |  |
| Frederico 2017 |  |  |  |  |
| Kirton 2011 |  |  |  |  |
| McMillen 2015 |  |  |  |  |
| ***Concurrent planning*** | | | | |
| Kenrick 2009 |  |  |  |  |
| Monck 2004 |  |  |  |  |

*Stabler is excluded as it did not evaluate the effectiveness of interventions

Yellow highlighted cells indicate primary studies included in each review.

# Appendix B AMSTAR-2 ratings of included systematic reviews

|  | **Q1** | **Q2*** | **Q3** | **Q4*** | **Q5** | **Q6** | **Q7*** | **Q8** | **Q9*** | **Q10** | **Q11*** | **Q12** | **Q13*** | **Q14** | **Q15*** | **Q16** | **Overall** |
| --- | --- | --- | --- | --- | --- | --- | --- | --- | --- | --- | --- | --- | --- | --- | --- | --- | --- |
| **Bai (2022)** | Y | N | N | PY | Y | N | N | PY | N | N | NA | NA | N | N | NA | Y | Critically low |
| **Bergin (2023)** | Y | Y | N | Y | N | N | N | PY | PY | N | NA | NA | Y | N | NA | Y | Low |
| **Bezeczky (2020)** | Y | PY | N | PY | Y | Y | N | N | Y | N | Y | Y | Y | Y | Y | Y | Low |
| **Carlson (2020)** | Y | PY | N | PY | Y | N | N | PY | PY | Y | NA | NA | N | N | NA | N | Critically low |
| **Davidson (2019)** | N | N | N | PY | N | N | N | N | N | N | NA | NA | N | N | NA | Y | Critically low |
| **Economidis (2023)** | Y | Y | N | Y | Y | N | N | Y | Y | N | NA | NA | Y | N | NA | Y | Low |
| **Goodwin (2020)** | Y | N | N | PY | N | N | N | PY | N | N | NA | NA | N | N | NA | N | Critically low |
| **Gregory-Wilson (2022)** | Y | N | N | PY | Y | N | N | PY | N | N | NA | NA | N | N | NA | Y | Critically low |
| **Holdroyd (2023)** | Y | Y | N | PY | Y | Y | N | PY | Y | N | NA | NA | N | N | NA | Y | Critically low |
| **Jaggi (2022)** | Y | N | N | PY | N | N | N | PY | N | N | NA | NA | N | N | NA | Y | Critically low |
| **Jones (2017)** | N | N | N | PY | N | N | N | N | N | N | NA | NA | N | N | NA | N | Critically low |
| **LaBrenz (2020)** | Y | N | Y | PY | Y | Y | N | PY | Y | N | NA | NA | Y | N | NA | Y | Critically low |
| **Landers (2016)** | Y | N | N | PY | N | N | N | N | N | N | NA | NA | N | N | NA | Y | Critically low |
| **Lee (2014)** | N | N | Y | PY | N | Y | N | N | N | N | NA | NA | N | Y | NA | Y | Critically low |
| **Lindner (2023)** | Y | N | N | PY | N | Y | N | PY | Y | N | NA | NA | N | Y | NA | Y | Critically low |
| **Lloyd (2018)** | Y | N | Y | PY | N | N | N | N | N | N | NA | NA | N | Y | NA | N | Critically low |
| **Marcellus (2017)** | Y | Y | N | Y | Y | Y | Y | N | N | N | NA | NA | N | N | NA | Y | Critically low |
| **Mason (2019)** | Y | PY | N | PY | N | N | N | PY | Y | N | NA | NA | N | Y | NA | Y | Critically low |
| **Meindl (2019)** | Y | N | N | PY | Y | Y | N | N | N | N | NA | NA | N | N | NA | N | Critically low |
| **Neo (2021)** | Y | PY | Y | PY | Y | N | N | PY | Y | N | Y | NA | Y | Y | NA | Y | Critically low |
| **NICE (2021)** | Y | Y | Y | Y | N | N | Y | Y | Y | Y | NA | NA | Y | Y | NA | Y | Moderate |
| **Noorishad (2020)** | Y | PY | N | PY | Y | N | N | PY | PY | N | NA | NA | N | N | NA | N | Critically low |
| **Nurmatov (2020)** | Y | Y | Y | Y | Y | Y | Y | Y | Y | N | NA | NA | Y | Y | NA | Y | Moderate |
| **Saeteurn (2022)** | Y | N | Y | PY | Y | N | N | Y | PY | N | NA | NA | N | N | NA | Y | Critically low |
| **Schaub (2022)** | Y | N | N | PY | N | N | N | PY | Y | N | NA | NA | N | N | NA | Y | Critically low |
| **Scott (2013)** | N | N | N | PY | N | N | N | N | N | N | NA | NA | N | N | NA | N | Critically low |
| **Sheehan (2018)** | Y | Y | Y | Y | Y | N | N | PY | Y | N | NA | NA | Y | Y | NA | N | Low |
| **Simkiss (2013)** | Y | N | N | PY | Y | N | Y | PY | Y | N | NA | NA | N | N | NA | Y | Critically low |
| **Stabler (2022)** | Y | N | N | PY | Y | Y | N | PY | N | N | NA | NA | N | N | NA | Y | Critically low |
| **van Assen (2020)** | Y | N | N | PY | Y | N | N | N | Y | N | Y | NA | Y | Y | Y | Y | Critically low |
| **White (2016)** | Y | N | Y | PY | N | N | N | N | N | N | NA | NA | N | N | NA | N | Critically low |
| **Wood (2022)** | Y | PY | N | PY | Y | N | N | PY | N | N | NA | NA | N | N | NA | Y | Critically low |
| **Zhang (2018)** | Y | N | Y | PY | N | Y | N | PY | N | N | Y | NA | N | Y | Y | Y | Critically low |
| Q1: Did the research question and inclusion criteria include the components of PICO?  Q2: Did it contain a statement that methods were established prior to the review and justify deviations to the protocol?  Q3: Did the review authors explain their selection of the study designs for inclusion in the review?  Q4: Did the review authors use a comprehensive literature search strategy?  Q5: Did the review authors perform study selection in duplicate?  Q6: Did the review authors perform data extraction in duplicate?  Q7: Did the review authors provide a list of excluded studies and justify the exclusions?  Q8: Did the review authors describe the included studies in adequate detail?  Q9: Did the review authors use a satisfactory technique for assessing the risk of bias (RoB) in individual studies that were included in the review?  Q10: Did the review authors report on the sources of funding for the studies included in the review?  Q11: If meta-analysis was performed did the review authors use appropriate methods for statistical combination of results?  Q12: If meta-analysis was performed, did the review authors assess the potential impact of RoB in individual studies on the results of the meta-analysis or other evidence synthesis?  Q13: Did the review authors account for RoB in individual studies when interpreting/ discussing the results of the review?  Q14: Did the review authors provide a satisfactory explanation for, and discussion of, any heterogeneity observed in the results of the review?  Q15: If they performed quantitative synthesis did the review authors carry out an adequate investigation of publication bias (small study bias) and discuss its likely impact on the results of the review?  Q16: Did the review authors report any potential sources of conflict of interest, including any funding they received for conducting the review? | | | | | | | | | | | | | | | | | |

Abbreviations: Y=Yes; N=No; PY=Partial Yes; NA=Not Applicable (no meta-analysis conducted)

*Indicates critical domains

# Appendix C: Example search strategy

**Database: Ovid MEDLINE(R) ALL <1946 to January 31, 2024> 01/02/2024**
**Search Strategy:**
**1**  systematic review.pt. (251298)
**2**  systematic$ review$.ti,ab. (322830)
**3**  meta-analysis as topic/ (23724)
**4**  meta-analytic$.ti,ab. (10178)
**5**  meta-analysis.ti,ab,pt. (282301)
**6**  metanalysis.ti,ab. (634)
**7**  metaanalysis.ti,ab. (1878)
**8**  meta analysis.ti,ab. (251596)
**9**  meta-synthesis.ti,ab. (1649)
**10**  metasynthesis.ti,ab. (471)
**11**  meta synthesis.ti,ab. (1649)
**12**  meta-regression.ti,ab. (13912)
**13**  metaregression.ti,ab. (977)
**14**  meta regression.ti,ab. (13912)
**15**  (synthes$ adj3 literature).ti,ab. (6311)
**16**  (synthes$ adj3 evidence).ti,ab. (18849)
**17**  integrative review.ti,ab. (5125)
**18**  data synthesis.ti,ab. (14955)
**19**  (research synthesis or narrative synthesis).ti,ab. (7774)
**20**  (systematic study or systematic studies).ti,ab. (15622)
**21**  (systematic comparison$ or systematic overview$).ti,ab. (4803)
**22**  evidence based review.ti,ab. (2468)
**23**  comprehensive review.ti,ab. (28457)
**24**  critical review.ti,ab. (20169)
**25**  quantitative review.ti,ab. (853)
**26**  structured review.ti,ab. (1050)
**27**  realist review.ti,ab. (657)
**28**  realist synthesis.ti,ab. (374)
**29**  or/1-28 (578175)
**30**  review.pt. (3272844)
**31**  medline.ab. (173655)
**32**  pubmed.ab. (227040)
**33**  cochrane.ab. (144459)
**34**  embase.ab. (166645)
**35**  cinahl.ab. (48275)
**36**  psyc?lit.ab. (917)
**37**  psyc?info.ab. (62869)
**38**  (literature adj3 search$).ab. (97956)
**39**  (database$ adj3 search$).ab. (103641)
**40**  (bibliographic adj3 search$).ab. (3980)
**41**  (electronic adj3 search$).ab. (35286)
**42**  (electronic adj3 database$).ab. (50414)
**43**  (computeri?ed adj3 search$).ab. (4038)
**44**  (internet adj3 search$).ab. (4438)
**45**  included studies.ab. (48364)
**46**  (inclusion adj3 studies).ab. (26936)
**47**  inclusion criteria.ab. (137917)
**48**  selection criteria.ab. (37249)
**49**  predefined criteria.ab. (2654)
**50**  predetermined criteria.ab. (1352)
**51**  (assess$ adj3 (quality or validity)).ab. (120039)
**52**  (select$ adj3 (study or studies)).ab. (95684)
**53**  (data adj3 extract$).ab. (104777)
**54**  extracted data.ab. (22024)
**55**  (data adj2 abstracted).ab. (6717)
**56**  (data adj3 abstraction).ab. (2391)
**57**  published intervention$.ab. (259)
**58**  ((study or studies) adj2 evaluat$).ab. (259883)
**59**  (intervention$ adj2 evaluat$).ab. (16252)
**60**  confidence interval$.ab. (584808)
**61**  heterogeneity.ab. (245559)
**62**  pooled.ab. (134149)
**63**  pooling.ab. (17229)
**64**  odds ratio$.ab. (381384)
**65**  (Jadad or coding).ab. (240241)
**66**  or/31-65 (2092906)
**67**  30 and 66 (365501)
**68**  review.ti. (733757)
**69**  68 and 66 (290461)
**70**  (review$ adj4 (papers or trials or studies or evidence or intervention$ or evaluation$)).ti,ab. (268525)
**71**  29 or 67 or 69 or 70 (891656)
**72**  letter.pt. (1242035)
**73**  editorial.pt. (680647)
**74**  comment.pt. (1030047)
**75**  72 or 73 or 74 (2223067)
**76**  71 not 75 (871828)

**77**  out of home.ti,ab,kf. (2025)
**78**  OoHC.ti,ab,kf. (67)
**79**  looked after.ti,ab,kf. (689)
**80**  Group Homes/ (1029)
**81**  Residential Facilities/ (5758)
**82**  Halfway Houses/ (1070)
**83**  group home$1.ti,ab,kf. (1044)
**84**  (congregate adj3 (care$ or home$1 or institution$ or placement$ or setting$ or accommodation$ or facilit$ or centre$ or center$ or unit$)).ti,ab,kf. (577)
**85**  (residential adj3 (care or home$1 or institution$ or placement$ or setting$ or accommodation$ or facilit$ or centre$ or center$ or unit$)).ti,ab,kf. (12671)
**86**  (institution$ adj3 (care or home$1 or placement$ or setting$ or accommodation$ or facilit$ or centre$ or center$ or unit$)).ti,ab,kf. (26164)
**87**  (supported accommodation$ or semi-independent accommodation$).ti,ab. (140)
**88**  (local authority adj3 (care or home$ or accommodation$)).ti,ab. (178)
**89**  ((state or public) adj care).ti,ab,kf. (408)
**90**  (guardian$ adj3 care).ti,ab,kf. (134)
**91**  (guardianship or special guardian$).ti,ab,kf. (1103)
**92**  (care adj1 order$).ti,ab,kf. (180)
**93**  placement order$.ti,ab,kf. (16)
**94**  care proceeding$.ti,ab,kf. (94)
**95**  "taken into care".ti,ab,kf. (70)
**96**  (remov$ adj6 (home$1 or family or families or parent$ or mother$ or father$ or mum$ or dad$)).ti,ab,kf. (3824)
**97**  77 or 78 or 79 or 80 or 81 or 82 or 83 or 84 or 85 or 86 or 87 or 88 or 89 or 90 or 91 or 92 or 93 or 94 or 95 or 96 (53094)
**98**  exp Child/ (2185758)
**99**  exp Infant/ (1266316)
**100**  Adolescent/ (2233928)
**101**  Young Adult/ (1021032)
**102**  Minors/ (2841)
**103**  (child$ or infant$ or infancy or baby or babies or newborn$ or toddler$ or preschool$ or pre school$).ti,ab,kf. (2217195)
**104**  (girl or girls or boy or boys or kid or kids).ti,ab,kf. (280055)
**105**  (adolescen$ or puberty or prepuberty or pubescen$ or prepubescen$ or teen$ or youth or youths or preteen$ or juvenil$).ti,ab,kf. (583622)
**106**  (young person$1 or young people or young adult$).ti,ab,kf. (163806)
**107**  (minor or minors).ti,ab,kf. (264023)
**108**  98 or 99 or 100 or 101 or 102 or 103 or 104 or 105 or 106 or 107 (5459696)
**109**  97 and 108 (14613)
**110**  ((child$ or infant$ or infancy or baby or babies or newborn$ or toddler$ or preschool$ or pre school$ or girl or girls or boy or boys or kid or kids or adolescen$ or puberty or prepuberty or pubescen$ or prepubescen$ or teen$ or youth or youths or preteen$ or juvenil$ or young person$1 or young people or young adult$) adj2 remov$).ti,ab,kf. (1480)
**111**  109 or 110 (15832)
**112**  76 and 111 (556)

**113**  Child, Institutionalized/ (1945)
**114**  ((child$ or infant$ or infancy or baby or babies or newborn$ or toddler$ or preschool$ or pre school$ or girl or girls or boy or boys or kid or kids or adolescen$ or puberty or prepuberty or pubescen$ or prepubescen$ or teen$ or youth or youths or preteen$ or juvenil$ or young person$1 or young people or young adult$ or minor or minors) adj2 in care).ti,ab,kf. (715)
**115**  childrens home$1.ti,ab,kf. (847)
**116**  ((residential or institution$) adj1 childcare).ti,ab,kf. (49)
**117**  (care adj1 (leaver$ or leaving)).ti,ab,kf. (160)
**118**  (exit$ adj2 care).ti,ab,kf. (255)
**119**  ((kinship or nonkinship or non kinship) adj3 care$).ti,ab,kf. (371)
**120**  ((connected or substitute$) adj2 care).ti,ab,kf. (432)
**121**  ((extraparental or extra parental) adj3 care).ti,ab,kf. (3)
**122**  Foster Home Care/ (3931)
**123**  Child, Foster/ (196)
**124**  (foster adj2 (care or home$1 or placement$ or setting$)).ti,ab,kf. (3673)
**125**  (foster adj2 (family or families or parent or parents or mother$ or father$ or mum$ or dad$ or carer$ or caregiv$ or care-giv$)).ti,ab,kf. (1872)
**126**  (foster$ adj2 (child$ or infant$ or infancy or baby or babies or newborn$ or toddler$ or preschool$ or pre school$ or girl or girls or boy or boys or kid or kids or adolescen$ or puberty or prepuberty or pubescen$ or prepubescen$ or teen$ or youth or youths or preteen$ or juvenil$ or young person$1 or young people or young adult$ or minor or minors)).ti,ab,kf. (2313)
**127**  Adoption/ (4935)
**128**  Child, Adopted/ (162)
**129**  (adopt$ adj2 (child$ or infant$ or infancy or baby or babies or newborn$ or toddler$ or preschool$ or pre school$ or girl or girls or boy or boys or kid or kids or adolescen$ or puberty or prepuberty or pubescen$ or prepubescen$ or teen$ or youth or youths or preteen$ or juvenil$ or young person$1 or young people or young adult$ or minor or minors)).ti,ab,kf. (3320)
**130**  Orphanages/ (489)
**131**  Child, Orphaned/ (782)
**132**  orphanage$.ti,ab,kf. (798)
**133**  (orphan$ adj2 (child$ or infant$ or infancy or baby or babies or newborn$ or toddler$ or preschool$ or pre school$ or girl or girls or boy or boys or kid or kids or adolescen$ or puberty or prepuberty or pubescen$ or prepubescen$ or teen$ or youth or youths or preteen$ or juvenil$ or young person$1 or young people or young adult$ or minor or minors)).ti,ab,kf. (896)
**134**  or/113-133 (19101)
**135**  76 and 134 (582)

**136**  112 or 135 (1038)

**137**  care-experienced.ti,kf. (48)
**138**  (care-experienced adj5 (child$ or infant$ or infancy or baby or babies or newborn$ or toddler$ or preschool$ or pre school$ or girl or girls or boy or boys or kid or kids or adolescen$ or puberty or prepuberty or pubescen$ or prepubescen$ or teen$ or youth or youths or preteen$ or juvenil$ or young person$1 or young people or young adult$ or minor or minors)).ti,ab,kf. (63)
**139**  (care-experienced adj3 (person$ or people or population$ or group$ or adult$ or parent$ or mother$ or father$ or mum$ or dad$)).ti,ab,kf. (74)
**140**  137 or 138 or 139 (148)
**141**  76 and 140 (18)

**142**  136 or 141 (1051)
**143**  exp animals/ not humans.sh. (5192574)
**144**  142 not 143 (1047) 1.	Bergin N, Turner F, Minnis H. The impact of infant mental health on legal decision making within the child welfare system: A systematic review. 2023;

2.	Anonymous. Interventions to support looked-after children and young people transitioning out of care to living with adoptive or birth parents or special guardians, or into connected care: Looked-After Children and Young People: Evidence review L. Review. *National Institute for Health and Care Excellence*. 10 2021;10:10.

3.	Bezeczky Z, El-Banna A, Petrou S, et al. Intensive family preservation services to prevent out-of-home placement of children: a systematic review and meta-analysis. *Child Abuse Negl*. 2020;102:104394. doi:<https://dx.doi.org/10.1016/j.chiabu.2020.104394>

4.	LaBrenz CA, Panisch LS, Liu C, Fong R, Franklin C. Reunifying successfully: a systematic review of interventions to reduce child welfare recidivism. *Res Soc Work Pract*. 2020;30(8):832–45. doi:<https://dx.doi.org/10.1177/1049731520936747>

5.	Holdroyd I, Barton G, Holdroyd D. The effect of working tax credits on child maltreatment rates: A systematic review. Systematic Review. *Child Abuse Negl*. 09 2023;143:106279. doi:<https://dx.doi.org/10.1016/j.chiabu.2023.106279>

6.	Wood S, Scourfield J, Stabler L, et al. How might changes to family income affect the likelihood of children being in out-of-home care? Evidence from a realist and qualitative rapid evidence assessment of interventions. Literature Review. *Children and Youth Services Review*. Dec 2022;143:1–11. doi:<https://dx.doi.org/10.1016/j.childyouth.2022.106685>

7.	Nurmatov UB, et a. *Impact of shared decision-making family meeting on children's out-of-home care, family empowerment and satisfaction: a systematic review*. What Works for Children's Social Care; 2020:118.
